# Noise reduction by upstream open reading frames

**DOI:** 10.1038/s41477-022-01136-8

**Published:** 2022-05-02

**Authors:** Ho-Wei Wu, Erickson Fajiculay, Jing-Fen Wu, Ching-Cher Sanders Yan, Chao-Ping Hsu, Shu-Hsing Wu

**Affiliations:** 1 Institute of Plant and Microbial Biology, Academia Sinica, Taipei, Taiwan; 2 Genome and Systems Biology Degree Program, Academia Sinica and National Taiwan University, Taipei, Taiwan; 3 Institute of Chemistry, Academia Sinica, Taipei, Taiwan; 4 Bioinformatics Program, Institute of Information Science, Taiwan International Graduate Program, Academia Sinica, Taipei, Taiwan; 5 Institute of Bioinformatics and Structure Biology, National Tsinghua University, Hsinchu, Taiwan; 6 Division of Physics, National Center for Theoretical Sciences, National Taiwan University, Taipei, Taiwan

**Keywords:** Plant molecular biology, Plant physiology

## Abstract

Gene expression is prone to burst production, making it a highly noisy process that requires additional controls. Upstream open reading frames (uORFs) are widely present in the 5′ leader sequences of 30–50% of eukaryotic messenger RNAs^1–3^. The translation of uORFs can repress the translation efficiency of the downstream main coding sequences. Whether the low translation efficiency leads to a different variation, or noise, in gene expression has not been investigated, nor has the direct biological impact of uORF-repressed translation. Here we show that uORFs achieve low but precise protein production in plant cells, possibly by reducing the protein production rate. We also demonstrate that, by buffering a stable TIMING OF CAB EXPRESSION 1 (TOC1) protein production level, uORFs contribute to the robust operation of the plant circadian clock. Our results provide both an action model and the biological impact of uORFs in translational control to mitigate transcriptional noise for precise protein production.

## Abstract (web summary)

Upstream open reading frames (uORFs) can repress translation efficiency to achieve low but precise protein production. Wu et al. show that uORFs function to buffer a stable production of a clock protein, TOC1, for sustaining a robust circadian rhythm in plant cells.

## Main

Gene expression is a sequential and highly coordinated process that conveys genetic information in genes (DNA) first to messenger RNAs (mRNAs) and then to functional protein products^4^. The dynamic and robust gene expression relies on multilayered regulation at the DNA, mRNA and protein levels. Bursts of de novo transcription and translation often result in fluctuations of both mRNA and protein levels and even lead to signal amplifications^5^. For specific genes, the temporal expression surges may be beneficial^5^, such as for cell fate determination in *Drosophila* embryogenesis^6^ or *Arabidopsis* sepal morphogenesis^7^. However, the ‘noisy’ production for most cellular mRNAs may be unproductive, energy consuming or even detrimental to the cells and organisms, especially if the fluctuating mRNAs encode key signalling molecules.

Noise inherited from transcription can be buffered by mRNA degradation^8^ and translational control. At the level of translation, one effective way to circumvent the negative impact of stochastic mRNA production is to have regulatory information built in *cis* in mRNAs for translational control. Such information can be the codon usage^9^, the presence of a secondary structure^10^ or microRNA target sites in eukaryotic mRNAs^11^. Another class of *cis* regulatory sequences is upstream open reading frames (uORFs) present in 5′ leader sequences of 30–50% of mRNAs in diverse organisms^1–3^. The translation of these uORFs can reduce the translation efficiency of the downstream main ORF (mORF) in a broad spectrum of eukaryotes, such as yeast^12^, human^13^, vertebrates^14^ and plants^15^. The prevalence of uORFs implies their instrumental roles in translation control. These observations motivated us to investigate whether uORFs can mitigate noisy transcription by reducing protein production capacity and variation, and, if so, how do uORFs exert their regulatory role in a biologically relevant context?

## Results

**Fig. 1:**
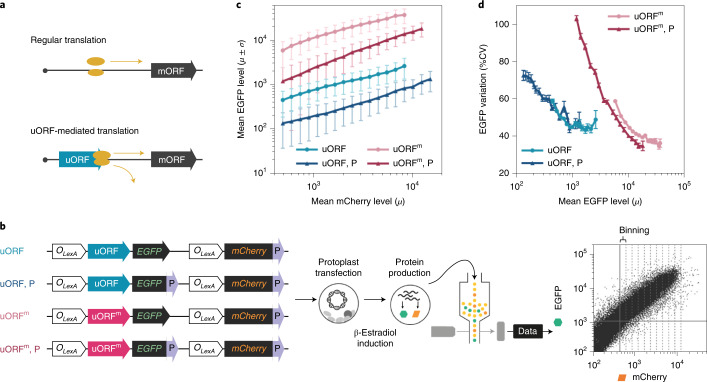
AUG-initiated uORF attenuates EGFP signal levels and noise. **a**, A sketch of regular or uORF-mediated translation via ribosomes (yellow ovals). **b**, Constructs with dual fluorescence reporters (EGFP and mCherry) under the control of inducible promoters (*O*_*LexA*_), with AUG-initiated or CUG-initiated uORF in the 5′ leader of native *EGFP* (solid circle, uORF and uORF^m^). P, PEST domain (solid triangle). **c**, Background-subtracted mean EGFP levels with s.d. (*μ* ± *σ*) across equal-distance mCherry measurement level bins (*n* = 15–18). Fluorescence was measured by flow cytometry. Three independent experiments were performed with replicates shown in Extended Data Fig. 1. Cell numbers are listed in Source Data Fig. 1. **d**, The association of mean EGFP signal levels and CV (%CV, *σ*/*μ*) in constructs indicated. Error bars represent s.d. (*σ*) from bootstrapping with 1,000 iterated resampling in **d**. Source data

We first examined protein production and variations in single *Arabidopsis* cells by comparing translation of mORFs in mRNAs with or without uORFs in their 5′ leaders. For an mRNA without uORF(s), ribosomes scan and assemble to translate the mORF (regular translation; Fig. 1a). For an mRNA with translatable uORF(s), translating ribosomes would dissociate after termination or resume scanning for translational re-initiation of the mORF^16^ (uORF-mediated translation; Fig. 1a). Although the CUG initiation codon is a common alternative initiation codon^17,18^, only an AUG-initiated, but not CUG-initiated, uORF could impose translation repression of the mORF in plants^15^ and yeast^19^. Figure 1b shows combinatorial constructs used to monitor the translation of mORF (enhanced green fluorescent protein (EGFP)) from mRNAs transiently produced in *Arabidopsis* protoplasts by estradiol induction^20^, under the control of an AUG-initiated uORF (uORF) or a CUG-initiated uORF (uORF^m^) in the 5′ leader sequences and with or without the fusion of a protein destabilization signal PEST domain (P)^21^. mCherry levels were used to infer the transcript levels in flow cytometry assays. We assumed the same transcription rate for *EGFP* and *mCherry* under the control of the identical promoters and the positive correlation of *mCherry* mRNA and protein level.

**Extended Data Fig. 1:**
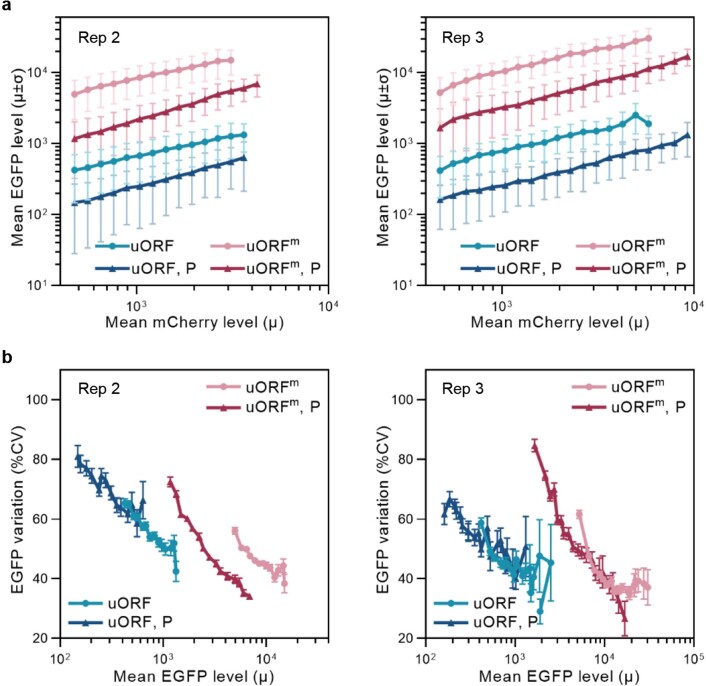
Replicate results showing that AUG-initiated uORF attenuates EGFP signal levels and noise. Background subtracted mean EGFP levels with SD (μ±𝜎) across equal-distance mCherry bins (**a**), and the coefficient of variation (%*CV)* vs mean EGFP levels (μ) (**b**) in constructs shown in Fig. 1c, **d**. Error bars represent standard deviations (𝜎) from bootstrapping with 1,000 iterated resampling in **b**. Results shown are replicates 2 and 3 of independent experiments from dual fluorescence assays (Fig. 1). Cell numbers are listed in Source Data_Fig. 1). Source Data

In uORF^m^, the fusion of PEST reduced the EGFP levels produced from transcripts spanning over a magnitude difference in abundance (indicated by mean mCherry level; uORF^m^, P versus uORF^m^ in Fig. 1c). The reduction in EGFP level is even more evident under the control of uORF (uORF versus uORF^m^; Fig. 1c and Extended Data Fig. 1a). In addition to the markedly reduced EGFP level, the coefficient of variation (CV, *σ*/*μ*) was also much lower for the uORF groups (Fig. 1d). The variations were negatively correlated with EGFP level for both the uORF and uORF^m^ groups (Fig. 1d and Extended Data Fig. 1b), similar to the observation by monitoring the expression variation of 43 yeast proteins under 11 experimental conditions^22^. The results supported our hypothesis that the uORF-mediated translational repression could effectively attenuate both the protein level and variation inherited from gene expression variabilities in single-cell populations.

**Extended Data Fig. 2:**
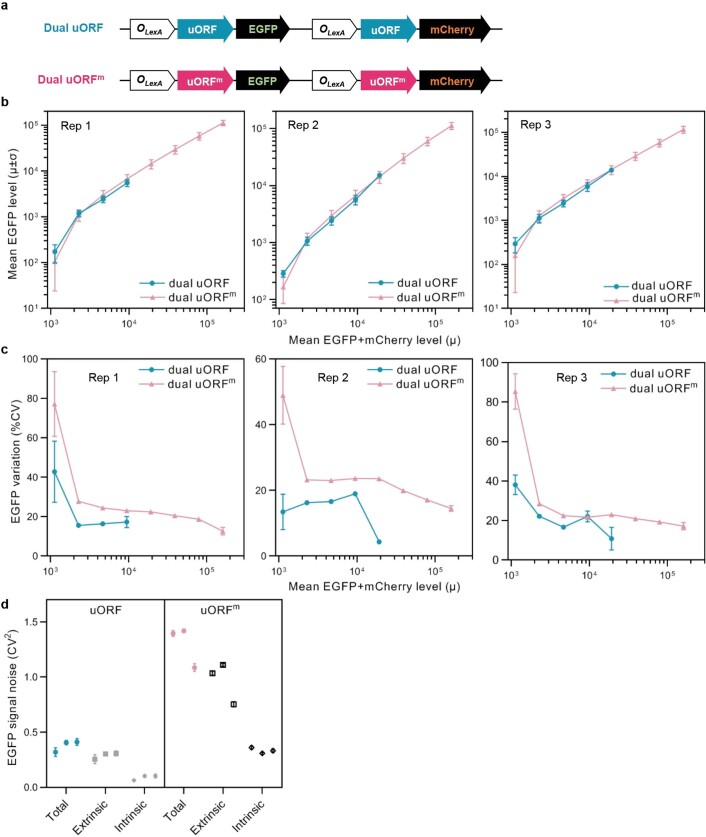
uORF-mediated translation represses mean signal level and variation in dual uORF/uORF^m^ constructs. **a**, Constructs with dual uORF/uORF^m^ in both fluorescence reporters (EGFP and mCherry) under the control of inducible promoters (*O*_*LexA*_), with AUG- or CUG-initiated uORF in the 5' leader of native *EGFP* and *mCherry*. **b**, Mean EGFP signal levels with SD (μ±𝜎) across equal-distance bins (n = 4-8) of mCherry+EGFP signal levels after background subtraction. Fluorescence was measured by flow cytometry. Three independent experiments performed. Cell numbers are listed (Source Data ED_Fig. 2). **c**, The association of mean EGFP signal levels and coefficient of variation (%*CV*, 𝜎/μ) in constructs indicated. Error bars represent standard deviations (𝜎) from bootstrapping with 1,000 iterated resampling. Error bars smaller than dots were not shown. **d**, The quantification of EGFP signal noise in *CV* square (●) from extrinsic (■) and intrinsic noise (◆) in dual fluorescence constructs expressed in cell populations for three independent experiments. Error bars represent standard deviations (𝜎) from bootstrapping with 1,000 iterated resampling. Source data

Inspired by the dissection of noise reported recently^11,23–25^, we also generated constructs with both EGFP and mCherry under the control of uORF or uORF^m^ to evaluate the impact on both intrinsic and extrinsic noise (Extended Data Fig. 2). The intrinsic noise indicates the amount of variation in one protein (for example, EGFP) that is independent of the other (mCherry), whereas the covariance of the two is essentially referred as the extrinsic noise. In principle, intrinsic noise reflects the variation in the expression process of a gene, and the extrinsic noise refers to the variation among cells, such as cellular resources^26,27^. However, factors in translation machineries could influence both intrinsic and extrinsic noise in our measurement scheme. The mathematical derivation of intrinsic and extrinsic noise is detailed in the Supplementary Information. The results confirmed that uORFs could effectively repress intrinsic as well as extrinsic noise by translational repression (Extended Data Fig. 2).

**Fig. 2:**
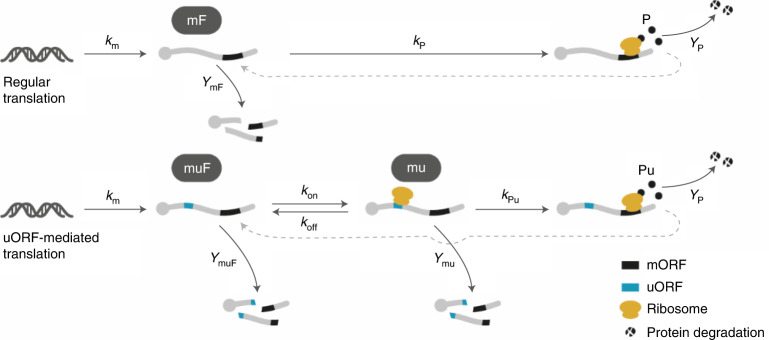
Reaction schemes for regular and uORF-mediated translation. Illustrations showing reaction species, directions and constants for regular and uORF-mediated translation for modelling. mF and muF, free-form mRNA state without and with uORF, respectively; mu, mRNA state with uORF occupied with ribosome; P and Pu, protein state from regular and uORF-mediated translation, respectively; Reaction rates for the production of mRNA (*k*_*m*_) and protein (*k*_p_ and *k*_pu_), the association of ribosome to muF (*k*_on_) or dissociation of the ribosome from mu (*k*_off_); degradation rates of mF (*γ*_mF_), muF (γ_muF_), and mu (*γ*_mu_)*k*_*x*_, *γ*_*x*_.

We developed a simple mathematical model to better understand the possible mechanism of the reduction in variation of protein levels in single cells. Mass action kinetics were built according to the schemes depicted, including a state of mRNA with uORF occupied with ribosome (mu) in the scenario (Fig. 2). With the linear noise approximation^28^, a set of mathematical expressions for the variances and covariances of species in the model under the number fluctuation caused by the probabilistic nature of the mass action (biochemical reactions), we fit experimental data for the parameters (detailed in Supplementary Information and Supplementary Table 1).

With the fitted model, the Fano factor (FF) for the uORF-regulated protein can be written as:

1 $${\mathrm{FF}}\left( {{\mathrm{Pu}}} \right) \equiv \frac{{{{{\mathrm{Var}}}}\left( {{\mathrm{Pu}}} \right)}}{{{\mathrm{Pu}}}} \cong 1 + \frac{{k_{ {\mathrm{Pu}}}}}{{\gamma _{{\mathrm{mu}}} + \gamma _{\mathrm{P}}}},$$

where Pu is the mean protein abundance under uORF-mediated translation, Var(Pu) is the corresponding variance, *k*_Pu_ is the protein production rate, *γ*_P_ and *γ*_mu_ are the degradation rates for the protein and mRNA, respectively, and mu denotes the mRNA with uORF occupied with ribosome. In Equation (1), the variation in protein is described by the intrinsic noise (1, the FF for a Poisson distribution) and the noise propagated from the upstream species, mu*.* Under the limit of higher degradation rate in mu (*γ*_mu_ >> *γ*_P_) the latter (~*k*_Pu_/*γ*_mu_) is approximately the mean amount of protein each mu generates before it degrades, a factor that contributes both the overall protein production and its variation. In the fitted parameters (Supplementary Table 1), the protein production rate is reduced by a factor of >6 (*k*_P_/*k*_Pu_) in the uORF regulation, whereas the degradation rate of mu remains similar to that of regular mRNA (*γ*_mu_ ≈ *γ*_mF_) detailed in Supplementary Fig. 2, thus indicating that the lower variation of uORF-regulated translation may result from reduced protein production rate. Therefore, by reducing the translational rates, uORFs act to reduce the uncertainty in the protein production.

Protein variations derived from stochastic gene expression affect steady-state proteins but also those functioning in key cellular pathways, such as cell cycle regulation^29^ and gene regulatory networks^30^. The oscillating nature of circadian genes also makes them more vulnerable to stochastic gene expression. To put the regulatory roles of uORFs in the biological context, we examined how uORFs contribute to the robust operation of the circadian clock in *Arabidopsis*.

Organisms on Earth have evolved to possess internal circadian clocks oscillating in ~24-hour cycles to synchronize with the external day/night cycles. The dyssynchronization of internal and external clocks often leads to disease or disadvantageous surviving fitness^31,32^. TIMING OF CAB EXPRESSION 1 (TOC1) is a key central oscillator of the *Arabidopsis* circadian clock^33,34^. Plants with increased TOC1 protein level have circadian period length >24 hours^35^ and slower cell cycle during vegetative development^36^, so an appropriate TOC1 protein level may be a prerequisite of optimal growth fitness.

**Extended Data Fig. 3:**
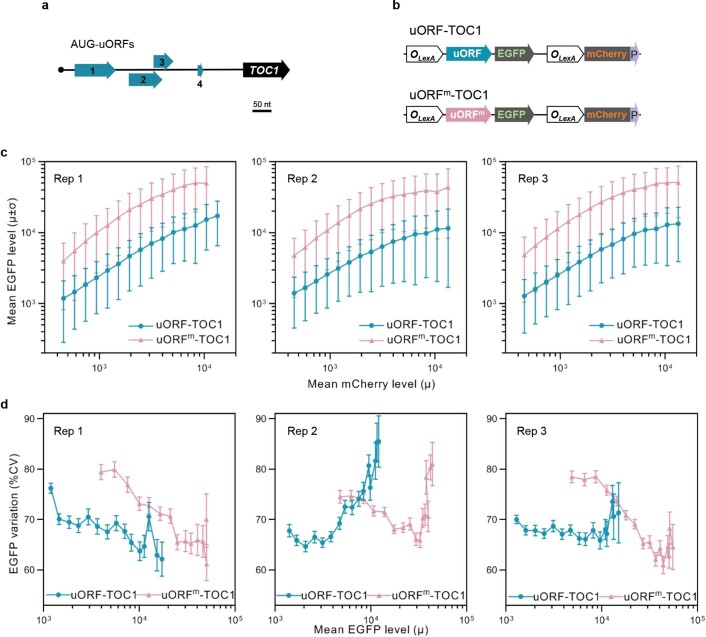
Triplicate results showing that uORFs of *TOC1* attenuate mean EGFP signals and variation in Arabidopsis protoplasts. **a**, Schematic illustration of the *TOC1* 5’ leader sequence carrying 4 AUG-initiated uORFs. **b**, Constructs with dual fluorescence reporters (EGFP and mCherry) under the control of inducible promoters (*O*_*LexA*_), with uORFs initiated with AUG or stop codons in the 5’ leader of *EGFP* (uORF-TOC1 and uORF^m^-TOC1, respectively). P: PEST domain. **c**, Mean EGFP levels with SD (μ±𝜎) across equal-distance mCherry bins (n = 14-15) after background subtraction. Fluorescence was measured by flow cytometry. Cell numbers are listed (Source Data_ED_Fig. 3). **d**, The association of mean EGFP levels and coefficient of variation (%*CV*, 𝜎/μ) in constructs indicated. Error bars represent standard deviations (𝜎) from bootstrapping with 1,000 iterated resampling. Source data

*TOC1* mRNA has four AUG-initiated uORFs in its 5′ leader sequence (Extended Data Fig. 3a). Ribosome protection fragments were identified for all four uORFs in a previously reported dataset^15^, thus suggesting active translation of these uORFs. We then assayed how the translation of these uORFs affects TOC1 protein production and the circadian robustness in plants. Mutations of AUGs to stop codons (TAG or TGA) (*uORF*^*m*^*–TOC1*; Extended Data Fig. 3b) led to increased level and variation of the reporter protein EGFP in *Arabidopsis* protoplasts (Extended Data Fig. 3c,d), which confirms that these uORFs have the potential to effectively buffer TOC1 protein production.

**Fig. 3:**
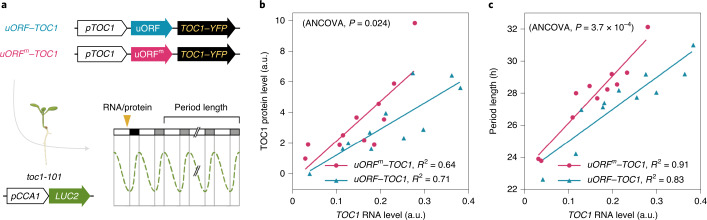
uORFs attenuate TOC1 protein production to sustain a robust circadian clock. **a**, Schematic illustration of constructs used to complement *toc1-101* mutant carrying the *pCCA1:LUC2* reporter and experimental design for measuring RNA/protein levels at ZT14 and circadian period lengths as described in Methods. **b**,**c**, Scatter dot plots showing correlations of *TOC1* RNA and TOC1–YFP protein levels (**b**) and circadian period length (**c**) in independent *uORF–TOC1* and *uORF*^*m*^*–TOC1* transgenic lines (*n* = 11). TOC1 RNA and protein levels are in arbitrary units (a.u.). One-way statistical analysis of covariance (ANCOVA) for two independent groups are provided in Source Data Fig. 3. Regression analysis indicated with *P* values and *R*^2^ values. Three independent experiments were performed and are shown Extended Data Fig. 7. Source data

**Extended Data Fig. 4:**
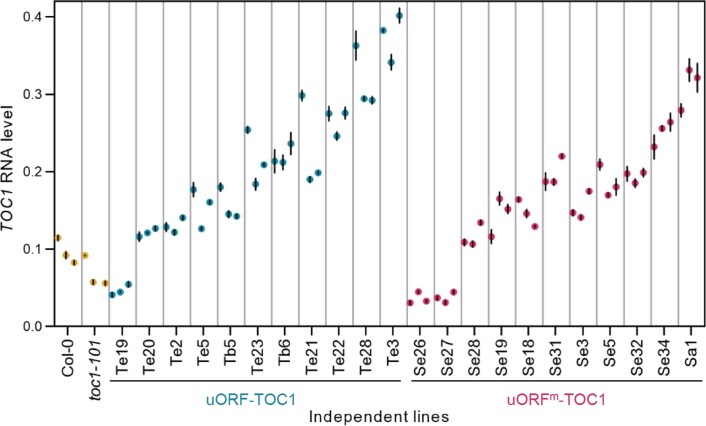
Triplicate results for *TOC1-YFP* mRNA levels in transgenic lines used. Scatter dot plot showing *TOC1-YFP* mRNA levels at ZT14 in 12-d-old T2 seedlings of uORF-TOC1 and uORF^m^-TOC1 lines used in this study (bulked n = 16-20). Dots and error bars show mean RNA values ±SD of three technical replicates with three independent quantifications. Results were normalized to *UBQ10* mRNA. Wild-type (Col-0) and *toc1-101*-carrying *pCCA1:LUC2* plants were included for comparison. Source data

We next examined whether uORFs in *TOC1* mRNA contribute to the translational control of TOC1 protein production in plants by complementing the *toc1-101* mutant with a *TOC1* mini-gene^35^ carrying the wild-type *TOC1* 5′ leader with uORFs or with each AUG mutated to a stop codon (*uORF–TOC1* and *uORF*^*m*^*–TOC1*, respectively; Fig. 3a). Independent transgenic lines with transgene mRNA levels spanning an order of magnitude (Extended Data Fig. 4) were selected to investigate the inter-relationship of *TOC1* mRNA, TOC1 protein and circadian period length (inferred by the *pCCA1:LUC2* reporter gene) (Fig. 3a).

**Extended Data Fig. 5:**
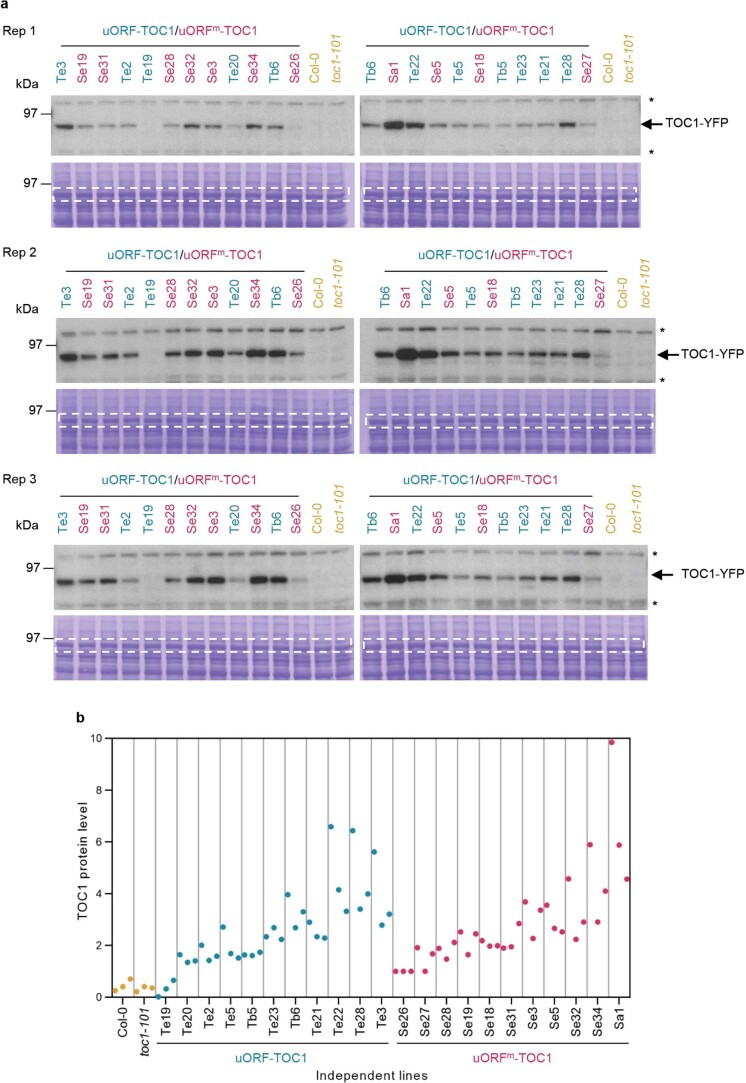
Triplicate results for TOC1-YFP protein levels in transgenic lines used. **a**, TOC1-YFP protein levels at ZT14 in 12-d-old T2 seedlings of uORF-TOC1 and uORF^m^-TOC1 lines used in this study visualized and quantified by immunoblotting with anti-GFP antisera. *: non-specific signals. Results from three independent quantifications are shown. **b**, Scatter dot plot showing TOC1-YFP protein levels normalized to Coomassie Blue-stained gels (boxed areas with dashed white lines in **a**). Wild-type (Col-0) and *toc1-101*-carrying *pCCA1:LUC2* plants were included for comparison. Source data

**Extended Data Fig. 6:**
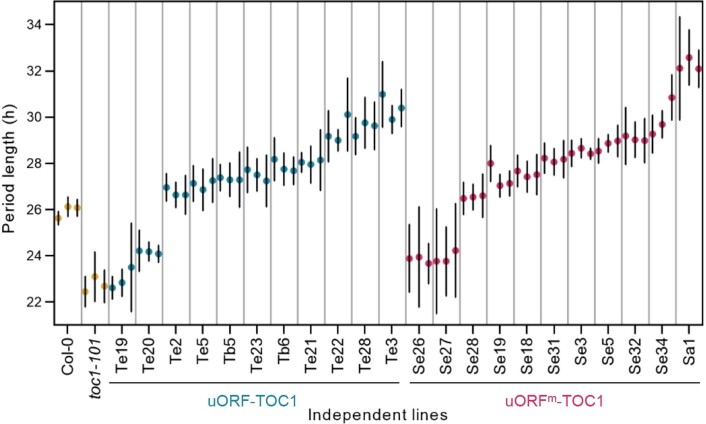
Triplicate results for period lengths in transgenic lines used. Scatter dot plots showing circadian period lengths of individual uORF-TOC1 and uORF^m^-TOC1 lines used in this study. Results were calculated from LUC2 data under continuous light (LL; 24-120 h). Three independent quantifications were performed. Dots and error bars show mean period length ±SD from a population of seedlings (n = 13-16 with RAE < 0.3) with three independent quantifications. Wild-type (Col-0) and *toc1-101*-carrying *pCCA1:LUC2* plants were included for comparison.

**Extended Data Fig. 7:**
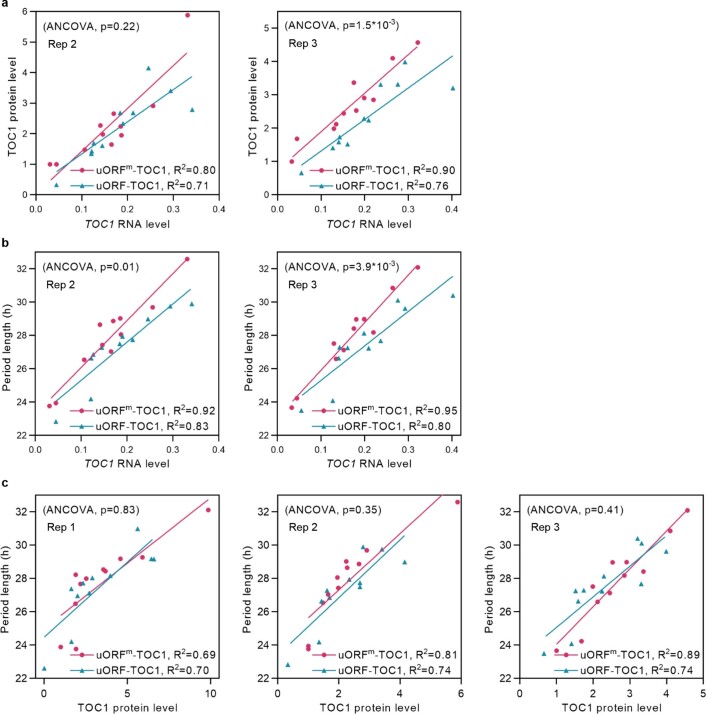
Replicate results showing that uORFs attenuate TOC1 protein production to sustain a 24-h circadian clock. Scatter dot plots showing correlations of *TOC1* RNA with TOC1-YFP protein levels (**a**), with circadian period lengths (**b**) in replicates 2 and 3 shown in Fig. 3b**, c**, and TOC1-YFP protein levels with circadian period lengths (**c**) in 3 biological replicates in uORF-TOC1 and uORF^m^-TOC1 lines (n = 11). Statistical analysis of covariance, one-way ANCOVA for 2 independent groups, and regression analysis indicated with p-values and R^2^ values.

At comparable mRNA levels, increased TOC1-yellow fluorescent protein (YFP) protein levels were produced from the transgene with *uORF*^*m*^*–TOC1* compared with *uORF–TOC1* (Fig. 3b and Extended Data Figs. 4, 5 and 7a). Therefore, *uORF*^*m*^*–TOC1* transgenic lines acquire significantly longer circadian periods than the *uORF–TOC1* lines (Fig. 3c and Extended Data Figs. 6 and 7b). These results suggest that uORFs function to confine the TOC1 protein level, which is crucial for plants to sustain a 24-hour circadian clock.

**Fig. 4:**
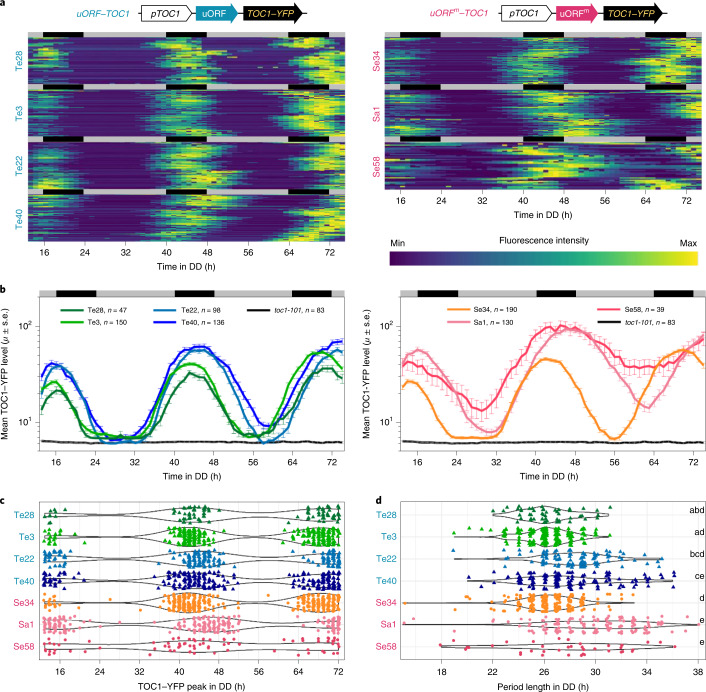
uORFs confine TOC1 protein level, phase and period length to achieve clock robustness in single cells. **a**, Heat maps showing time course (hours in continuous dark, DD) of mean TOC1–YFP level in nuclei of cells isolated from representative *uORF–TOC1* and *uORF*^*m*^*–TOC1* lines. Horizontal lines are individual cells ranked by *k*-means clustering. Scaled fluorescence intensity from minimum to maximum shown in the pseudo-colour bar. **b**, Mean ± s.e.m. of TOC1–YFP protein levels through time. Individual lines marked in different colours with number (*n*) of cells analysed. Black line, *toc1-101* negative cells. **c**, Violin plots of curve-fitted peak phase distribution of individual cells from each line through time (hours). **d**, Violin plots of period length distribution defined by successive curve-fitted peaks. Both the first and/or the second period length for each line were plotted. Letters denote statistically significant subgroups (two-tailed *F*-test, *P* < 0.001). Three independent experiments were performed and are shown in Extended Data Figs. 9 and 10. Data used are listed in Source Data Fig. 4. Source data

**Extended Data Fig. 8:**
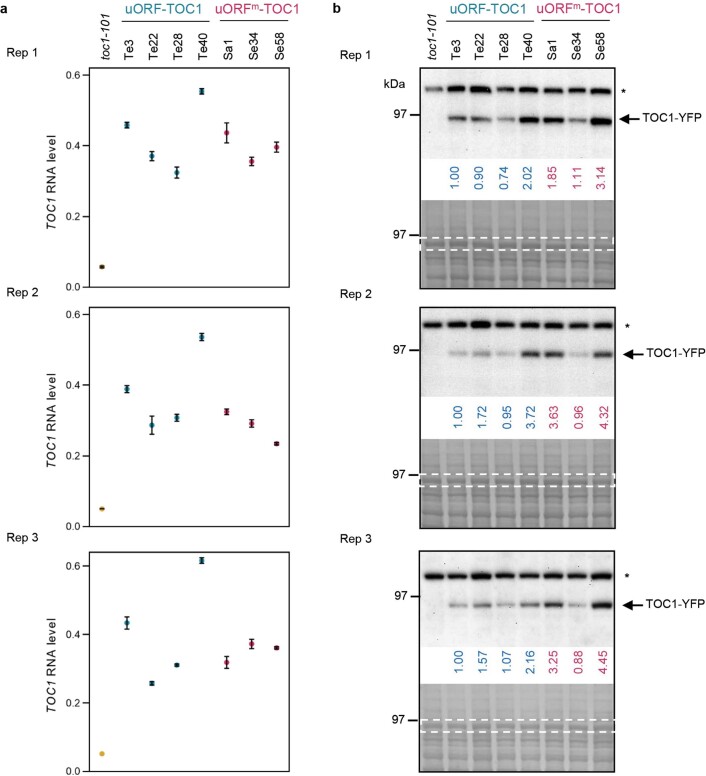
Triplicate results showing *TOC1-YFP* RNA and protein levels in representative lines used for single cell experiments in Fig. 4. **a**, *TOC1-YFP* mRNA levels at ZT14 in 12-d-old T2 seedlings of uORF-TOC1 and uORF^m^-TOC1 lines used in this study. Data and error bars show as mean RNA values ±SD of three technical replicates with three independent quantifications. Results were normalized to the level of *UBQ10* mRNA. **b**, TOC1-YFP protein levels at ZT14 in 12-d-old T2 transgenic seedlings of uORF-TOC1 and uORF^m^-TOC1 lines indicated were visualized and quantified by immunoblot with anti-GFP antisera. *: non-specific signals. Numbers indicated TOC1-YFP protein levels normalized to Coomassie Blue-stained gels (boxed areas with dashed white lines) with TOC1-YFP level in Te3 set to 1. *toc1-101*-carrying *pCCA1:LUC2* plants was included for comparison. Results from 3 biological replicates are shown. Source data

**Extended Data Fig. 9:**
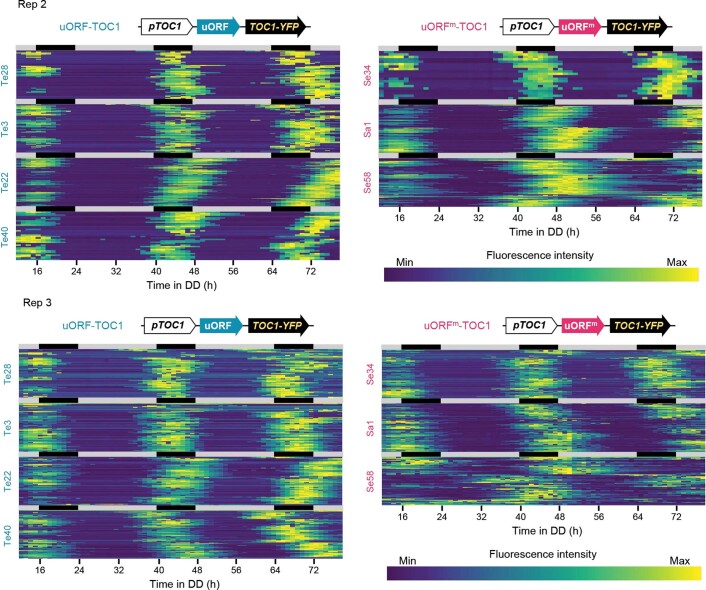
Replicate results showing that cellular rhythms are more robust in uORFs cells. Results are replicates 2 and 3 of single cell time-course experiments shown in Fig. 4a. Heatmaps showing time-course (h in continuous dark, DD) mean TOC1-YFP level in nuclei of cells isolated from representative uORF-TOC1 and uORF^m^-TOC1 lines. Horizontal lines are individual cells ranked by k-means clustering. Scaled fluorescence intensity from minimum to maximum shown in the pseudo-colour bar. Data used were listed in Source Data Fig. 4. Source data

**Extended Data Fig. 10:**
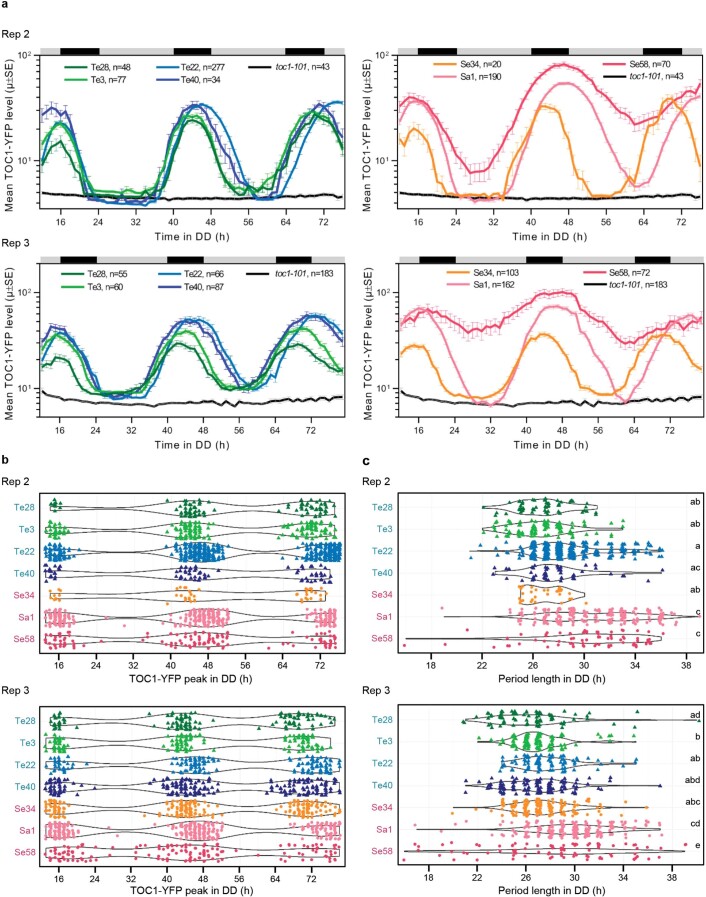
Replicate results showing that uORFs confine TOC1-YFP protein expression phase and period length. Results are replicates 2 and 3 of single cell time-course experiments shown in Fig. 4b, **c, d**. **a**, Mean TOC1-YFP protein levels through time. Error bar as standard error of measurements. **b**, Violin plots of curve-fitted peak phase distribution of individual cells from each line through time (h). **c**, Violin plots of period length distribution defined by successive curve-fitted peaks. Letters denote statistically significant subgroups (two-tailed F-test, p < 0.001). Both the first and/or the second period length for each line were plotted. Letters denote statistically significant subgroups (two-tailed F-test, p < 0.001). Data used were listed in Source Data Fig. 4. Source Data

The clock behaviour in a whole plant is a combined result of cell-to-cell, even organ-to-organ, communication^37,38^. We next examined whether uORFs also exert regulatory roles in TOC1 protein production and clock precision at the level of the individual cells. A time-course quantification of nucleus-localized TOC1–YFP fusion protein in cells isolated from seven independent lines was conducted (four for *uORF–TOC1* and three for *uORF*^*m*^*–TOC1*) (Extended Data Fig. 8). The results showed more synchronized circadian oscillation of TOC1–YFP in individual cells from *uORF–TOC1* lines than in *uORF*^*m*^*–TOC1* cells with comparable TOC1–YFP levels (Fig. 4a,b and Extended Data Figs. 8, 9 and 10a). The peak phases are less coherent and period lengths broadly distributed for the *uORF*^*m*^*–TOC1* cells (Fig. 4c,d and Extended Data Fig. 10b,c). These data supported that, without functional uORFs in the 5′ leader, TOC1–YFP oscillations are much noisier among cells, thus compromising the clock robustness in individual cells.

In the *Arabidopsis* circadian clock, *TOC1* and *CCA1* represent evening and morning genes, respectively, and mutually inhibit the expression of each other, thus constituting a double negative feedback loop^34,39^. Variable TOC1 expression phases and periods cannot ensure the expression precision of the morning gene *CCA1*, which tips the balance of the *Arabidopsis* circadian clock. These results together support that uORF-repressed translation is required for plant cells to maintain an optimal and precise TOC1 protein level to sustain a robust circadian clock.

## Conclusions

In this Letter, we demonstrate that uORFs can effectively reduce both the intrinsic and extrinsic noise from stochastic gene expression (Extended Data Fig. 2). Protein production and variation are both attenuated by uORFs in individual cells expressing a wide range of mRNA levels (reporter constructs in Fig. 1 and the endogenous plant gene *TOC1* in Fig. 4). This is primarily achieved by the reduced protein production rate (Supplementary Table 1) in uORF-mediated translation. We also show that uORFs are vital for TOC1 protein accumulation with precision to sustain a robust clock in *Arabidopsis* (Figs. 3c and 4). Although circadian clocks in plants and animals both operate via feedback loops, they harbour distinct core clock components. Analogous to *TOC1* in the plant clock, the translation of some core clock genes in mice (*Arntl*, *Clock*, *Cry1*, *Nr1d1* and *Nr1d2*) was previously found to be regulated by uORFs^40^. Whether uORFs also contribute to the clock robustness in animals is worth investigating.

uORFs have been implicated to function in diverse physiological processes in eukaryotes, such as metabolite homoeostasis in plants^41–43^ and the association with human disease^44,45^. uORFs are also frequently observed in genes encoding protein kinases and transcription factors^3,46^. Studying whether uORFs function to ensure stable and precise signalling cascades potentiated by these signalling molecules would be of great interest.

The homoeostasis of a physiological response in a multicellular organism could be achieved by cell–cell coupling^47^. To maintain protein homoeostasis within a single cell, the use of uORFs in translation control demands a relatively lower energy budget, such as the passive reduction of noise by cellular compartmentation^48^, compared with routes via mRNA and/or protein degradation or using integral feedbacks that often require extrinsic biomolecules^49,50^. Hence, uORFs represent one significant mode of translational control, functioning cooperatively with additional regulatory mechanisms to ensure an effective and meticulous protein translation with low energy expenditure.

## Methods

### Constructs for protoplast transient assays

For dual fluorescence reporter assays (Fig. 1), DNA fragments harbouring *LexA–VP16–ER (XVE)*-inducible *LexA operon–35S* promoter and *T*_*Nos*_–*O*_*LexA*_–*MCS–T*_*3A*_ were PCR amplified from *pER10–Bar*^51^ and used to substitute the *35S* promoter and *Nos* terminator in *p326 35S:smGFP* vector to generate *p326 O*_*LexA*_–*smGFP–T*_*Nos*_*–O*_*LexA*_*–MCS–T*_*3A*_. *smGFP* was later replaced by *EGFP* from *pCAMBIA1390 35S:**FD-MCP-EGFP*. The mCherry coding sequence from *p326 35S:mCherry* was subcloned into MCS to create *p326 O*_*LexA*_–*EGFP–ZmSP–T*_*Nos*_*–O*_*LexA*_*–mCherry–ZmSP–T*_*3A*_. The maize *SPERMINE SYNTHASE 1* PEST sequence (*ZmSP*)^21^ was fused in-frame with *EGFP* or *mCherry* as a protein destabilization signal. DNA fragments harbouring the 5′ leader sequence of *AT3G55520* (*AtFKBP20-1*) carrying an AUG-uORF or a site-directed mutagenized CUG-uORF were inserted into the 5′ end of *EGFP* for single uORF/uORF^m^ constructs (Fig. 1 and Extended Data Fig. 1) and into both the 5′ ends of *EGFP* and *mCherry* for dual uORF/uORF^m^ constructs (Extended Data Fig. 2).

For translation activity quantification of uORFs in *AT5G61380* (*TOC1*) (Extended Data Fig. 3), the wild-type *TOC1* 5′ leader (*uORF–TOC1*) was amplified from genomic DNA. The four AUG-uORFs in *TOC1* 5′ leader were point mutated into stop codons (ATG^131^→TAG, ATG^255^→TGA, ATG^329^→TAG, and ATG^464^→TAG) by overlapping PCR and termed *uORF*^*m*^*–TOC1*. *uORF–TOC1* and *uORF*^*m*^*–TOC1* were subcloned separately into the 5′ end of *EGFP* in *p326 O*_*LexA*_*–EGFP–ZmSP–T*_*Nos*_*–O*_*LexA*_–*mCherry–ZmSP–T*_*3A*_. All primers used are listed in Supplementary Table 2.

### Dual fluorescence assays in *Arabidopsis* protoplasts

Transgenic plants expressing XVE transcription activator^52^ were established by transforming wild-type *Arabidopsis thaliana* (Col-0) with *pER10-Bar* vector^51^ with *Agrobacterium tumefaciens* strain GV3101. Successful expression of XVE protein was confirmed by immunoblotting with antiserum against LexA (Abcam, ab14553,1:10,000 dilution). Rosette leaves of 4-week-old transgenic plants grown under 12-hour light/dark at 22 °C were used for protoplast preparation as described^53^ by adopting a sandwich method^54^ and the use of W5 medium with 5 mM glucose for protoplast suspension. Each transfection involved 3.0 × 10^5^ protoplasts and 60 μg of plasmid DNA prepared by using Plasmid Maxi kits (QIAGEN). *p326 35S:EGFP* and *p326 35S:mCherry* were included as controls. XVE was induced to express in protoplasts by 2 μM β-estradiol for 16–40 hours. The quantification of EGFP (excitation 488 nm, emission 530/30 nm), mCherry (excitation 561 nm, emission 620/15 nm) and chlorophyll autofluorescence (excitation 488 nm, emission 695/40 nm) was accomplished by using the Attune NxT Flow Cytometer and software version 2.7 and version 3.1 (Thermo Fisher Scientific) and FlowJo version 10.

mCherry levels were classified by equal-distance binning in log-space for mean mCherry levels. The mean EGFP levels (*μ*) and standard deviation (*σ*) were then calculated for each mCherry bin after background subtraction. The 1,000 bootstrapping iterations of 50% subsampling were used to evaluate the standard deviation of the coefficient of variation (*σ*/*μ*) sampled.

For RNA levels and half-life measurements of uORF/uORF^m^-regulated *EGFP* transcripts (Supplementary Figs. 2 and 4), transfected protoplasts were induced to express the transgene by 16-hour β-estradiol treatment, followed by treatment with 10 μM actinomycin D for 1, 2 and 4 hours before cells were collected and used for RNA preparation. The NucleoSpin RNA XS column (Macherey-Nagel) was used for RNA isolation, followed by reverse transcription with SuperScript IV reverse transcriptase (Thermo Fisher Scientific) and quantitative PCR (qPCR) as described^55^. Primers used in this study are in Supplementary Table 2.

### Generation and characterization of *uORF–TOC1* and *uORF*^*m*^*–TOC1* transgenic plants

A DNA fragment harbouring an *uORF*^*m*^*–TOC1* leader sequence was used to replace the *uORF–TOC1* leader sequence in *pZP221 TMG–YFP*^35^ to create *pZP221* *TMG (uORF*^*m*^*)–YFP*. Primers used to generate *uORF*^*m*^–*TOC1* are in Supplementary Table 2. *TMG (uORF)–YFP* and *TMG (uORF*^*m*^*)–YFP* in binary vectors were introduced separately into the *toc1-101* mutant carrying the *pCCA1:LUC2* reporter gene^56^ by *Agrobacterium* GV3101.

For protein and RNA analyses (Fig. 3b,c and Extended Data Figs. 4, 5, 7 and 8), 12-day-old seedlings from T2 generations of *TMG (uORF)–YFP* and *TMG (uORF*^*m*^*)–YFP* transgenic seedlings grown on 1/2 Murashige and Skoog medium (MS) with gentamycin under 16-hour light/8-hour dark conditions were collected at Zeitgeber time 14 (ZT14). Total RNA was extracted with the pine-tree method^57^ and quantified by qPCR as described^55^ with QuantStudio 12K Flex software (version 1.2.2 for data acquisition and version 1.4 for data analysis) except for the primers used for *TOC1* in Supplementary Information Table 2. Total protein was extracted in freshly prepared extraction buffer^58^ (100 mM Tris-HCl, pH 7.8, 4 M urea, 5% SDS, 15% glycerol, protease inhibitor cocktail and 5% β-mercaptoethanol) and separated with NuPAGE Bis-Tris 4–12% gel. TOC1–YFP protein was detected by immunoblot analyses with anti-GFP antisera (GenLab, catalogue number 50005-05, 1:10,000 dilution) by Amersham Hyperfilm ECL (GE Healthcare) for SuperSignal (Thermo Fisher Scientific) linear chemiluminescence signal detection. Coomassie Brilliant Blue (Sigma-Aldrich)-stained membranes were loading controls. Data were analysed by using the Fiji_win64 image processing package^59^ (Extended Data Fig. 5). For Extended Data Fig. 8, immunoblot quantitation was performed by using UVP ChemStudio Plus Touch (Analytik Jena) for capturing linear chemiluminescence signals, and VisionWorks v9.1.20063.7760 was used for data acquisition and analysis.

For calculating the circadian period length (Fig. 3c and Extended Data Fig. 7), 7-day-old seedlings were transferred to 96-well black plates with 1/2 MS medium containing 0.5 mM luciferin (PerkinElmer). A light-proof chamber (Phenotron, HiPoint) with a SOPHIA 2048BX Large Format CCD Camera (Princeton Instruments) with LightField v6.10 was used to measure bioluminescence under constant light condition for 4 days (LL; 24–120 hours). A custom-made software, ImagePro v6.2, was used to acquire data from images for fast Fourier transform-nonlinear least squares (FFT-NLLS) analysis with the Biological Rhythms Analysis Software System (BRASS v3.0)^60^.

For measuring TOC1–YFP levels in single cells (Fig. 4 and Extended Data Figs. 9 and 10), TMG (uORF)–YFP and TMG (uORF^m^)–YFP transgenic plants were grown on 1/2 MS with gentamycin under 16-hour light/8-hour dark and 22 °C. Rosette leaves of 4-week-old plants were used to prepare protoplasts as described above. Protoplasts were recovered in W5 medium for 48 hours under an entrainment condition, transferred to darkness and imaged by Leica Application Suite X_4.1.0.23081 using STELLARIS 8 (Leica) confocal microscopy for YFP signals (excitation 488 nm, emission 518–581 nm) hourly at times corresponding to ZT12 to ZT80. Image analyses and quantification of TOC1–YFP signals in nuclei are described in ‘Analysis for single-cell trajectories’ in Supplementary Information. The raw data were used for generating heat maps, and averaged oscillation curves are shown in Fig. 4a,b and Extended Data Figs. 9 and 10a. Curved fitted data used for peak identification and period length calculation and violin plots were prepared with ggplot2 package version 3.3.5 in R x64 4.0.3 in Fig. 4c,d and Extended Data Fig. 10b,c.

### Reporting Summary

Further information on research design is available in the Nature Research Reporting Summary linked to this article.

## Supplementary information

Supplementary Information Supplementary Discussion, Figs. 1–6, and Tables 1 and 2.

Reporting Summary

## Data Availability

Source data are provided with this paper.

## Source data

Source Data Fig. 1 Statistical source data for Fig. 1 and Extended Data Fig. 1.
Source Data Fig. 3 Statistical source data for Fig. 3 and Extended Data Fig. 7.
Source Data Fig. 4 Statistical source data for Fig. 4, and Extended Data Figs. 9 and 10.
Source Data Extended Data Fig. 2 Statistical source data for Extended Data Fig. 2.
Source Data Extended Data Fig. 3 Statistical source data for Extended Data Fig. 3.
Source Data Extended Data Fig. 4 Statistical source data for Extended Data Figs. 4–6.
Source Data Extended Data Fig. 5 Unprocessed blots and gels for Extended Data Fig.5a.
Source Data Extended Data Fig. 8 Statistical source data for Extended Data Fig. 8.
Source Data Extended Data Fig. 8 Unprocessed blots and gels for Extended Data Fig. 8b.

## Code availability

All codes used in this study are available at https://github.com/cherrihsu/uORF_dataprocess.
