# Supplementary material for: Noise reduction by upstream open reading frames
Source: Nat Plants. 2022 May 2;8(5):474–80. doi: 10.1038/s41477-022-01136-8 (PMC9122824; doi:10.1038/s41477-022-01136-8)
Supplement: Supplementary file 1 — Supplementary Discussion, Figs. 1–6, and Tables 1 and 2. [file 41477_2022_1136_MOESM1_ESM.pdf]

nature portfolio

https://doi.org/10.1038/s41477-022-01136-8

Supplementary information

Noise reduction by upstream open reading frames

In the format provided by the authors and unedited

Supplementary Information

Regular gene expression

To recapitulate the molecular behavior of translational regulations, especially protein production level and variation, we introduced mathematical models for analytical studies. For regular gene expression, we adopted a previously described model[1,2] as a comparison for further modelling. Regular translation can initiate from free-form RNA as the following mass action kinetics:

$$\frac{d[mF]}{dt} = k_m - \gamma_{mF}[mF] \, , \qquad (1)$$

$$\frac{d[P]}{dt} = k_P[mF] - \gamma_P[P] \, . \qquad (2)$$

The ordinary differential equations (ODEs) denote the changes in level (denoted with square brackets) of the molecular species x (x = P, protein, or mF, free-form mRNA) over time ($\frac{d[x]}{dt}$). k_x is the corresponding production rate of species; γ_x is the degradation rate. Free-form mRNA, mF, can be produced with a constant transcription rate (k_m) and subjected to a first-order degradation ($\gamma_{mF}[mF]$). Protein, P, can be produced with translation, a term directly proportional to the concentration of free-form mRNA $k_P[mF]$, and subjected to a first-order degradation ($\gamma_P[P]$). In the regular translation model, free-form mRNA is engaged in translation for protein production without additional limitations. In this model, the mRNA under translation is neglected.

With a mathematical model, there are several approaches to quantify the fluctuations arising from the probabilistic nature of chemical reactions. For example, the chemical master equations (CMEs) describe the time-dependent probabilities of finding various abundance in all the components of the system[3], which cannot be solved analytically for many problems but stochastic trajectories can be numerically propagated accordingly[4]. From a first-order expansion of CMEs, the time change of the mean levels of the components follow the original mass action kinetics (the ODEs)[5]. Variances and covariances for the abundance of the components of the system can be obtained by a second-order expansion to the CMEs. With their linear scaling property to the system size, the linear noise approximation (LNA) can be derived[6,7], which is a set of ODEs describing the time change in the variances and covariances, expressed in terms of the reaction fluxes and their derivatives (Jacobian). The detailed expression can be found in previous reports[6,7]. From a set of ODEs with mass action kinetics, following the LNA scheme leads to analytical expressions for the variances and covariances, and the steady-state condition was adopted through this work.

The reaction equations of the regular translation model can be addressed accordingly and solved. At steady state, we have

$$[mF]_{ss} = \frac{k_m}{\gamma_{mF}}, \text{ and} \tag{3}$$

$$[P]_{ss} = \frac{k_m k_P}{\gamma_{mF}\gamma_P}. \tag{4}$$

Under stochastic considerations, the reactions take place with probabilistic events. At steady state, the mean protein and mRNA levels are the same as the steady-state levels

$$\langle mF \rangle = [mF]_{ss};\ \langle P \rangle = [P]_{ss}, \tag{5}$$

and the variance for mRNA at the steady-state can be obtained with LNA.

$$Var(mF) = \frac{k_m}{\gamma_{mF}}. \tag{6}$$

The variance for protein can also be obtained with LNA,

$$Var(P) = \frac{k_m k_P (k_P + \gamma_{mF} + \gamma_P)}{\gamma_{mF}\gamma_P(\gamma_{mF} + \gamma_P)} = [P]\left(1 + \frac{k_P}{\gamma_{mF}+\gamma_P}\right). \tag{7}$$

With the expression in equations (6,7), the square of coefficient of variation (CV, $\frac{\sigma}{\mu}$) for protein level P is

$$CV^2(P) = \frac{Var(P)}{[P]^2} = \frac{1}{[P]} + \frac{1}{[mF]}\frac{\gamma_P}{\gamma_{mF} + \gamma_P} \tag{8}$$

$$= \frac{\gamma_{mF}\gamma_P}{k_m k_P} + \frac{\gamma_{mF}}{k_m}\frac{\gamma_P}{\gamma_{mF}+\gamma_P}, \tag{9}$$

where the expression in equation (8) includes a term for intrinsic noise ($1/[P]$) and a term for extrinsic noise, which is the intrinsic noise of the mRNA ($1/[mF]$) scaled by the time-averaging factor ($\gamma_P/(\gamma_{mF} + \gamma_P$)) as described[8]. Although a little redundant, the expression in equation (9) is useful in evaluating the *CV*.

uORF-mediated translational regulation

We constitute the translational regulation by specifying translational initiation in uORF-containing mRNA. The uORF-containing mRNA can be separated into two species: the free-form mRNA with uORF, *muF*, and one with uORF occupied by ribosome, *mu*, with the presence of the ribosome association on the uORF. Only *mu* is allowed to

engage in translation of the main coding sequence for protein production. We could also assign different degradation rates to them as γ_{mu} and γ_{muF}. Following the model depicted in Fig. 2, the mathematical model for uORF-mediated translation can be written as the following mass action kinetics:

$$\frac{d[muF]}{dt} = k_m - k_{on}[muF] + k_{off}[mu] + k_{Pu}[mu] - \gamma_{muF}[muF] \,, \quad (10)$$

$$\frac{d[mu]}{dt} = k_{on}[muF] - k_{off}[mu] - k_{Pu}[mu] - \gamma_{mu}[mu] \text{ , and} \quad (11)$$

$$\frac{d[Pu]}{dt} = k_{Pu}[mu] - \gamma_P[Pu] \,. \quad (12)$$

In its scanning on mRNA, a ribosome would first encounter and bind to uORF, producing a uORF-occupied mRNA, mu, with association rate k_{on}. The ribosomes could reinitiate for protein production (with translation rate k_{Pu}) or dissociate from the uORF (with dissociation rate k_{off}), and both lead to free-form mRNA muF. Protein $[Pu]$ can be produced with translation, at a rate that is directly proportional to the concentration of uORF-occupied mRNA ($k_{Pu}[mu]$), and it is subjected to the first-order degradation ($\gamma_P[Pu]$). Upon protein production, the ribosome releases; therefore, this portion of mRNA goes back to its free form muF.

Here we note that, in the regular translation model, an mRNA does not disappear due to translation, and therefore there is no need to add an additional $k_P[mF]$ in equation (1). However, in uORF-regulated translation, an mRNA needs to go through the "mu" state before it can be translated and released back to the pool of muF, and therefore an explicit term $k_{Pu}[mu]$ is required.

The ODEs describing the reactions of uORF-mediated translation are solvable with LNA. In this model, an interconvertible loop between the muF and mu states is included. The steady-state concentrations for the mRNA species can be deduced as

$$[muF]_{ss} = \frac{k_m(k_{off} + k_{Pu} + \gamma_{mu})}{A} \text{ , and} \quad (13)$$

$$[mu]_{ss} = \frac{k_m k_{on}}{A} \text{ , where} \quad (14)$$

$$A = k_{off}\gamma_{muF} + k_{on}\gamma_{mu} + k_{Pu}\gamma_{muF} + \gamma_{muF}\gamma_{mu} \,. \quad (15)$$

For the protein steady-state concentrations, it is

$$[Pu]_{ss} = \frac{k_m k_{on} k_{Pu}}{(\gamma_P A)} = [mu]_{ss}\frac{k_{Pu}}{\gamma_P} \,. \quad (16)$$

Under stochastic dynamics, the mean protein and mRNA abundance are

$$\langle mu \rangle = [mu]_{ss};$$
$$\langle muF \rangle = [muF]_{ss}; \quad (17)$$
$$\langle Pu \rangle = [Pu]_{ss}.$$

The protein variance with uORF-mediated translation would be

$$Var(Pu) = \frac{k_m k_{on} k_{Pu}(\mathrm{B} + k_{on} k_{Pu})}{\mathrm{A}} = \langle Pu \rangle \left(1 + \frac{k_{on}\, k_{Pu}}{\mathrm{B}}\right), \text{ where} \quad (18)$$

$$\mathrm{B} = (\gamma_{muF} + \gamma_P)(k_{off} + k_{Pu}) + (\gamma_{mu} + \gamma_P)(k_{on} + \gamma_{muF} + \gamma_P). \quad (19)$$

Under the uORF-mediated translation control, the square of *CV* for the protein fluctuation is

$$CV^2(Pu) = \frac{1}{\langle Pu \rangle} + \frac{1}{\langle mu \rangle} \frac{k_{on}\gamma_P}{\mathrm{B}} \quad (20)$$

$$= \frac{1}{\langle Pu \rangle} + \frac{\mathrm{A}}{k_m} \frac{\gamma_P}{\mathrm{B}}, \quad (21)$$

with parameters A and B defined in equations (15, 19).

In addition to the mean and variance of the mRNA and protein species, we further deduce the covariance ($Cov_{(x,y)}$) between species from LNA. In uORF-mediated translation, we found an intriguing result:

$$Cov_{(muF,mu)} = 0. \quad (22)$$

There is a zero covariance between the amount of muF and mu in equation (22). Our model with interconversion between the free from and uORF-occupied mRNA implies a decoupling of the two mRNA pools.

Other relevant models for uORF-mediated regulation

Here, we considered a simple re-initiation model for inferring the possible noise-buffering mechanism. Various mechanisms can exist on uORF-mediated regulations, such as leaky scanning, ribosome stalling on uORF, and mRNA degradation in a uORF-occupied state, as reviewed previously[9]. Although desirable to explore the capacity of noise-reduction effects of these different mechanisms, most of the mathematical representations are similar to the re-initiation model, with small changes in the reactions, and the effects on noise reduction can be very similar.

Although leaky scanning separates uORF translation and mORF translation[10], the existence of uORF translation with another round of translation initiation would also provide feedback to stabilize the free-form mRNA, similar to the concept of the uORF-occupied mRNA state as a buffering pool in one our proposed models. The only difference would be leaky scanning with translation taking place from free-form mRNA (*muF*), instead of the reinitiated *mu*. This mechanism switches the source of translation, but the buffering system remains the same. Another possibility could be ribosome stalling on uORF, which would re-initiate downstream mORF translation later, regarded as a re-initiation model that can be described with our current model as longer accumulation in the *mu* state. In contrast, the repression effect for the uORF-mediated RNA degradation comes from the non-sense mediated decay (NMD) in the first run of translation in uORF[11]. As a futile transcription without protein production in mORF, this is a totally different regulatory mechanism that is beyond the scope of the present study. Moreover, if a repression of the protein production is from a reduced number of available mRNA, it alone is not likely sufficient to reduce the variation, and another potentially undiscovered mechanism likely plays a role.

Therefore, regardless of details including DNA copy number, ribosome availability, number of uORFs, ribosome stalling on uORF, transcript length and translation elongation rates, the model could adequately present the properties and the simplicity of uORF-mediated translational regulation.

Parameter estimation of the reaction constants in the models

With the regular translation model (equations 1-2) and the uORF-mediated translation model (equations 10-12) listed above, we computed the protein mean value and Fano factor (σ^2/μ) from LNA, and the results were fitted, aiming to reproduce the mean EGFP level and the Fano factor from the flow cytometry data. The flow cytometry data for the dual fluorescence system consist of 2 and 4 constructs (see below), respectively, with 3 replicates each. Fitting is done and the best parameter set that can reproduce the data is used. From fluorescence signals to the number of molecules, we measured fluorescence of AcGFP flow cytometer calibration beads (Takara) corresponding to number of Molecules of Equivalent Soluble Fluorochrome, and in this way we calibrated the fluorescence levels of proteins into their molecule numbers in the parameter estimation (Supplementary Fig. 1).

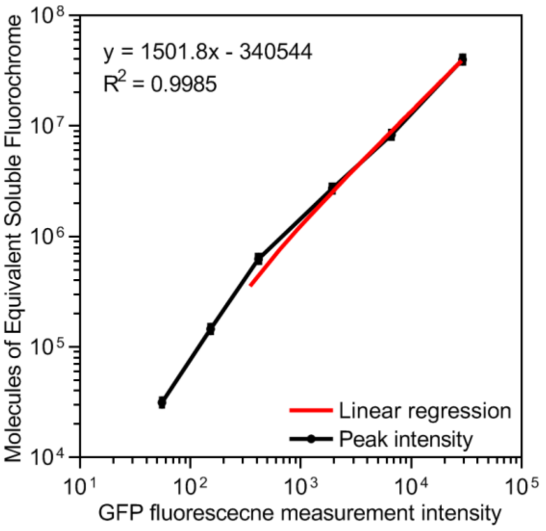

Supplementary Fig. 1 | Regression of GFP fluorescence measurement intensity with the Molecules of Equivalent Soluble Fluorochrome.

Parameter estimation was performed using the Metropolis-Hasting algorithm[12,13] combined with expectation maximization[14-16]. This approach is known as the Monte Carlo Expectation Maximization (MCEM)[17,18]. We implemented a simple MCEM scheme for our purpose. The likelihood function we used is the joint probability distribution of the error between true value and estimated value, which we assume to be independent and follow a normal distribution. A uniform prior was assumed. The log-likelihood of this function becomes the negative of the sum of the squared error between the protein mean value and Fano factor of experimentally derived levels and estimated values from LNA expression. Rate constants were drawn from a log-normal distribution, and with each draw, protein mean value and Fano factor estimates were calculated. In MCEM, the posterior probability is calculated at each sampling stage. In the maximization step, the ratio of posterior probability between consecutive draws (or the exponential of the difference between consecutive log-likelihood) are used to decide whether to accept or reject the latest values of the rate constant based on a uniform random variable. After several samplings (decided programmatically in the algorithm), the mean and standard deviation of the rate constant from all the accepted values are calculated and used as the mean and standard deviation for the next sampling stage. This serves as the expectation step in our implementation because we do not have a close form for the rate constants. The cycle is repeated until the calculated mean of the rate constants and standard deviations of each rate constants are no longer changing or until the maximum number of steps is reached. To initiate the process, we instantiate 5 separate chains (parallel runs) of MCEM while tracking and recording best rate constant values. The number of chains and number of steps grows as needed during run time to a maximum of 20 chains and 750000 steps. The computed values with the maximum posterior probability in each chain are recorded. The final set of parameters are chosen

from the top 5 chains with the minimum sum of squared error based on biological intuition.

In the optimization of parameters, protein and mRNA degradation half-lives are used [($t_{1/2}$ for EGFP protein=26 h (Ref[19]), $t_{1/2}$ for *EGFP* mRNA=2.5 h measured in this study (Supplementary Fig. 2)] together with background-corrected, molecule calibrated protein and noise levels.

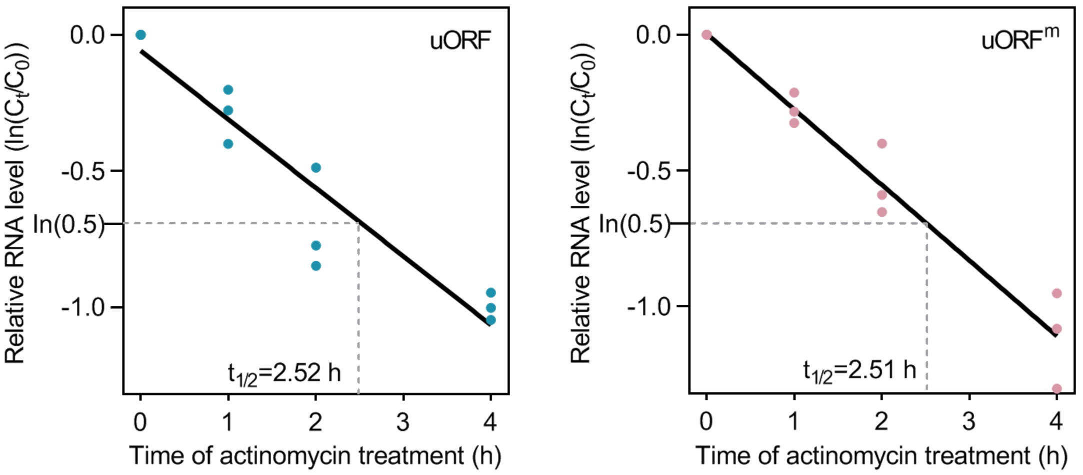

Supplementary Fig. 2 | Half-lives ($t_{1/2}$) of *EGFP* RNA under the control of uORF or uORF^m was determined by treating with actinomycin D for 1, 2, 4 h as described in Methods. Sample size (n=3). Regression test R^2>0.9 for regression lines.

For the system of constructs depicted in Fig. 1, the optimization is constrained based on four separate constructs in the experimental design (Fig. 1b). The following are the set of constraints we imposed in the process; the production of mRNA with k_m the same for all constructs, the degradation of mRNA $\gamma_{mF} = \gamma_{muF}$ the same for all constructs, γ_P the same for normal protein degradation with and without uORF (γ_P) and the same for the two PEST- associated proteins (γ_{P-PEST}), k_P the same for regular translation and k_{Pu} the same for uORF-mediated translation. The constraints above are incorporated in the objective function (posterior probability) to ensure that the values are meaningful to the experimental design.

Parameter sets of biological replicates are listed (Supplementary Table 1). The estimated parameters can recapitulate the mean protein levels and variance in the experimental data (Fig. 1c and Extended Data Fig. 1a).

Inferring possible mechanisms from the estimated parameters

With the fitted parameters, we note that among the first-order fitted rates, k_{on} and k_{Pu} are always much larger than k_{off}, γ_{mu}, γ_{muF}, and γ_P. Also, k_{on} is always larger than k_{Pu}.We could further simplify the expressions by

$$\mathrm{B} \approx (\gamma_{mu} + \gamma_P)k_{on} \,. \tag{23}$$

The CV^2 as in equation (20) becomes

$$CV^2(Pu) \approx \frac{1}{\langle Pu \rangle} + \frac{1}{\langle mu \rangle} \frac{\gamma_P}{(\gamma_{mu} + \gamma_P)}\,, \tag{24}$$

and equation (14) can be simplified

$$\langle mu \rangle \approx \frac{k_m}{\gamma_{mu}}\,, \tag{25}$$

which is very similar to the corresponding terms in regular translation, equations (3) and (8). In equations (8) and (24), the first term is the intrinsic noise for the level of protein. The second term resembles propagated noise, originating from the intrinsic noise of the upstream ($1/\langle mF \rangle$ or $1/\langle mu \rangle$), scaled by the time-averaging factor[4]. The result in equation (24) shows that the CV^2 of protein translated under uORF regulation follows a similar behavior for the regular expression, except now it follows the amount and the degradation rate of *mu*.

In the numerical fitting for the level of protein and the corresponding variances, the degradation rate for *mu* was similar to the values of γ_{muF} and γ_{mF}, the degradation rates for the free mRNA under uORF or the regular mRNA without uORF regulation. Namely, with $\gamma_{muF} \approx \gamma_{mF}$, $\langle mF \rangle$ is also very similar to $\langle mu \rangle$, and the CV^2 in equations (9) and (24) are very similar. In this case, the uORF-regulated protein level $\langle Pu \rangle$ is less than that for regular translated protein $\langle P \rangle$, and it is produced with a similar CV value. It is already a less varying situation, as seen in Fig. 1d of the main text: both sets of uORF and $\mathrm{uORF^m}$ data points are in a similar range in the %CV scale, but when extrapolating the trend of $\mathrm{uORF^m}$ data to low expression level, the %CV would be much higher than that for uORF. Here we can further analyze this situation with the Fano factor (FF):

$$FF(Pu) \equiv \frac{\mathrm{Var}(Pu)}{\langle Pu \rangle} \cong 1 + \frac{k_{Pu}}{\gamma_{mu}+\gamma_P}\,. \tag{26}$$

The corresponding FF for regular translation can be obtained from equation (7),

$$FF(P) \equiv \frac{\mathrm{Var}(P)}{\langle P \rangle} \cong 1 + \frac{k_P}{\gamma_{mF}+\gamma_P}\,. \tag{27}$$

With much lower translation rate k_{Pu} than k_P in the fitted model, a lower FF in the uORF regulated translation is seen. Therefore, the repression of translation rate reduces the variation in protein level in this model. Under the limit of higher degradation rates for the mRNA species $\gamma_{mF}, \gamma_{mu} \gg \gamma_p$, the FF becomes

$$FF \cong 1 + b, \tag{28}$$

where b is the burst size in translation[20,21], defined as k_{Pu}/γ_{mu} or k_P/γ_{mF}, the number of protein molecules produced before each mRNA is degraded. In other words, this model generates less variation in protein levels by reducing the translational burst size.

The possibility of small γ_{mu}

The exact mechanism of uORF regulation remains to be discovered, but we have always kept alternative hypotheses in mind as the research progresses. By fitting a population of heterogeneous cells with a set of uniform parameters in a simple model, the fitted result offers possible insights, and there can be other possibilities. One interesting aspect is on the degradation rate for *mu*: the mRNA state *mu* is with ribosome associated to its uORF, and thus, it is possibly more stable and degrade slower than the ribosome-free *mF* and *muF*. In fact, with the many trials and tests in the present study, a solution with $\gamma_{mu} \ll \gamma_{mF}, \gamma_{muF}$ can be obtained if the variation of protein levels is lower. If so, then $\langle mu \rangle$ is higher, leading to lower fluctuation, and thus the noise propagated to the protein $\langle Pu \rangle$ becomes lower. This is a case in which mRNA is stabilized through the *mu* state, which acts as a "buffer" to reduce the noise (Supplementary Fig. 3).

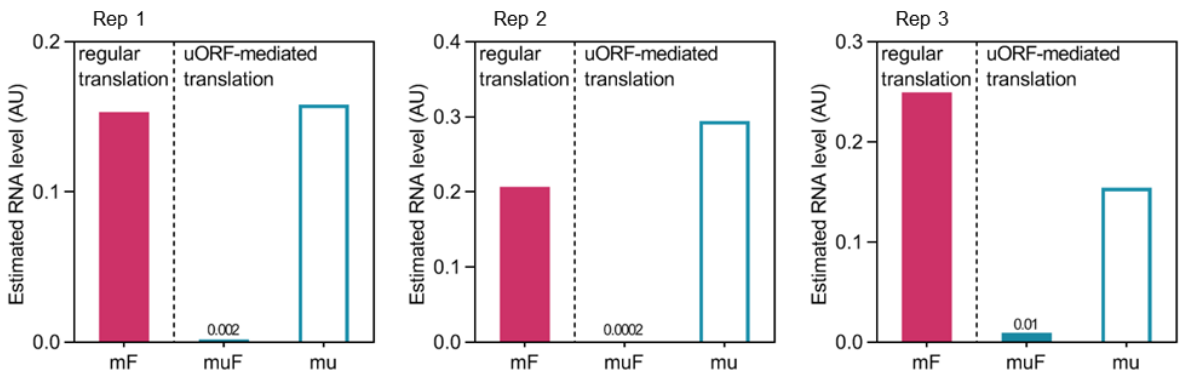

Supplementary Fig. 3 | RNA level in arbitrary units (AU) for *mF*, *muF* and *mu* by fitting the triplicated experimental data in Fig. 1 and Extended Data Fig. 1.

In this case, the longer mRNA lifetime and higher amount for mu should be expected. However, the results in Supplementary Fig. 4 showed comparable relative *EGFP* mRNA levels from uORF and uORF^m constructs (or slightly higher for uORF^m constructs), so do similar mRNA degradation rates for both (Supplementary Fig. 2). We note there is still a chance that the mRNA under translation is the predominant mRNA species because the translation machinery is excessive[22]. Also, in experiments, it is presently impossible to quantify the specific mRNA states. Therefore, these experimental results cannot be used to totally reject this hypothesis either.

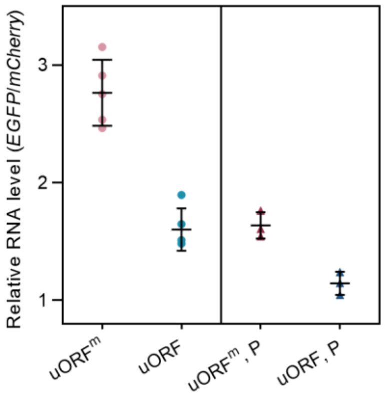

Supplementary Fig. 4 | *EGFP* RNA level relative to *mCherry* RNA level in protoplast transient assays conducted in Fig. 1. Constructs labeled represent those in Fig. 1. Sample size for native protein (n=5), and for PEST-labeled protein (n=3).

Nevertheless, the current conclusion, uORF-mediated reduction in protein production, and therefore reduction of the burst size in translation, is quite general and plausible. The possibility of having smaller γ_{mu} and a buffered pool of *mu* remains an open question. In this model, uORF can be viewed as a source of hindrance, such that fewer ribosomes can be recruited to translate downstream mORF. This is in line with ribosome profiling, where less ribosome binding is observed in the translation on uORF-containing transcripts[23]. Therefore, uORF regulation leads to lower protein production via smaller translation burst size, which also decreases gene expression noise.

Analysis of the fluorescent protein in flow cytometry data

Flow analysis and gating strategy

Gated target cells, mesophyll protoplasts, with forward scatter (FSC-H) and side scatter (SSC-H) channels, were used as the starting cell population (Supplementary Fig. 5a). We did not perform doublets removal because the cells were individual protoplasts. A

small amount of fluorescence is detected in the negative control, possibly from the autofluorescence of cells (Supplementary Fig. 5b). Fluorescence Minus One (FMO) controls as EGFP-only transfection (Supplementary Fig. 5c) or mCherry-only transfection (Supplementary Fig. 5d) were measured separately. The mCherry threshold was critically defined to allow a percentage (0.1%) of EGFP-only cells falling to the right in Attune NxT Software v2.7.0 for each experimental replicate. Fluorescence compensation was not conducted because of no noticeable cross-talks between the two fluorescence signals. mCherry-positive cells were subjected to data analyses (Supplementary Fig. 5e). The cell viability was supported by the presence of chlorophyll autofluorescence (Ex 488 nm, and Em 695/40 nm) in cells (Supplementary Fig. 5f). The pre-determined FSC-H/SSC-H gating and mCherry fluorescence thresholds were used for processing experimental data collected.

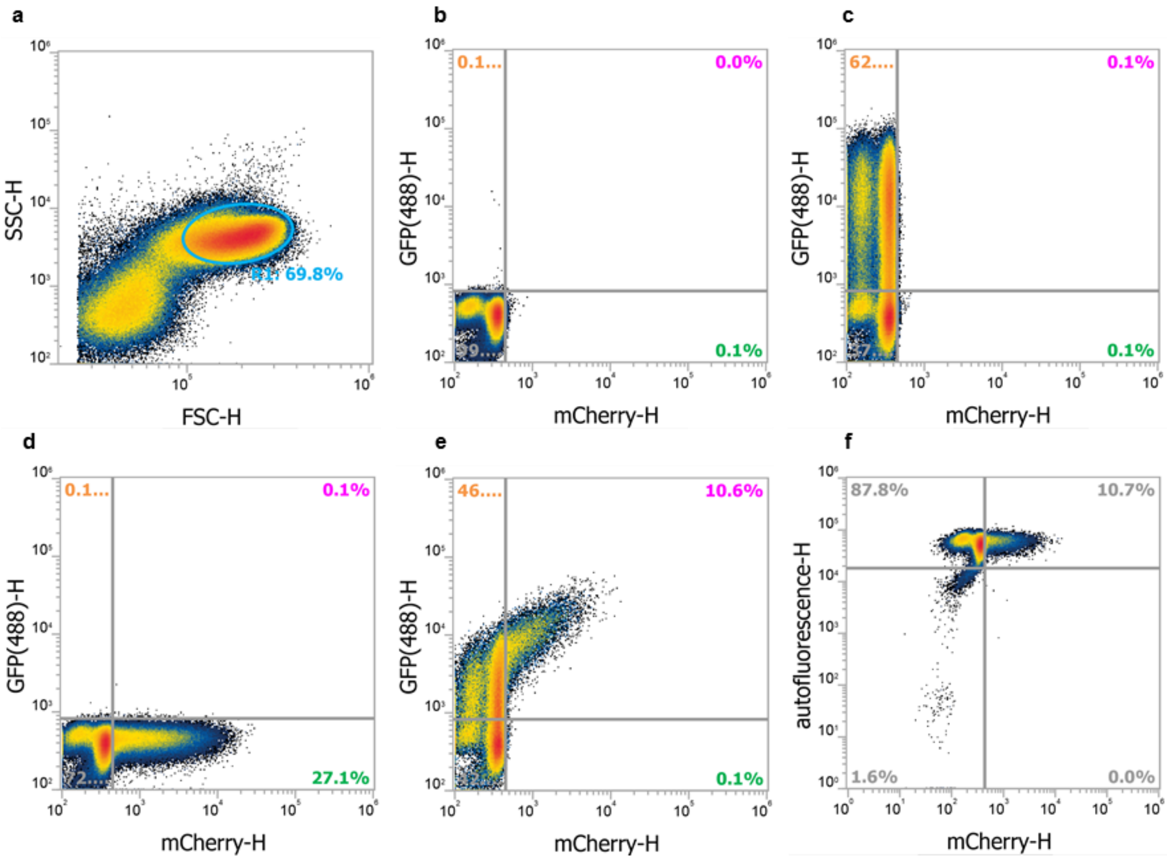

Supplementary Fig. 5 | Flow analysis and gating strategy for mesophyll population to quantify mCherry-H (561 nm) and EGFP-H (488 nm) fluorescence intensity. **a,** Forward scatter (FSC-H) and side scatter (SSC-H) gating (light-blue oval) of targeted mesophyll population. **b**, Cellular background measured in non-transfected cells. **c**, Subsetting gating of mCherry based on EGFP-only transfection as a Fluorescence-Minus One (FMO) control. A defined percentage (0.1%) falling to the right sets the mCherry threshold. **d**, mCherry-only transfection as an FMO control. **e,** Cell population (uORF^{m}) with positive signals for mCherry and EGFP are shown as an example. **f**, Chlorophyll autofluorescence signals indicate the cell viability.

Background subtraction

To remove background for the fluorescent signals obtained from flow cytometry, we followed the detailed description in a previous report[1]. Namely, the measurement from the flow cytometry *t* is considered as the sum of a real signal *s* and a background θ

$$t = s + \theta. \tag{29}$$

Here *t*, corresponds to the actual measurement intensity, which can be from EGFP (*t*) or from mCherry (t'). The mean of signal *s* is

$$\langle s\rangle = \langle t\rangle - \langle \theta\rangle, \tag{30}$$

where statistics for θ are obtained from the background cells measured in parallel in each experiment.

The variation for signal *s* is

$$\mathrm{Var}(s) = \mathrm{Var}(t) - \mathrm{Var}(\theta) - 2\mathrm{Cov}(s,\theta). \tag{31}$$

We followed the previous study[1] with the assumption that the signals *s* and background θ are correlated exclusively through cell size ω,

$$\mathrm{Cov}(s,\theta) = \langle s\rangle\langle\theta\rangle\eta_\omega^2. \tag{32}$$

For evaluating the noise contribution from *w*, η_ω^2, we took the signal of a channel that does not detect the protein, FSC-H (ϕ), and proceeded with

$$\mathrm{Cov}(t,\phi) = \mathrm{Cov}(\theta,\phi) + \mathrm{Cov}(s,\phi). \tag{33}$$

With a similar relationship of $\mathrm{Cov}(s,\phi) = \langle s\rangle\langle\phi\rangle\eta_\omega^2$, we have

$$\eta_\omega^2 = \frac{\mathrm{Cov}(t,\phi) - \mathrm{Cov}(\theta,\phi)}{\langle s\rangle\langle\phi\rangle}. \tag{34}$$

The total noise of *s*, η_s^2 can be represented as

$$\eta_s^2 = \left(\frac{\langle t\rangle}{\langle s\rangle}\right)^2 \eta_t^2 - \left(\frac{\langle\theta\rangle}{\langle s\rangle}\right)^2 \eta_\theta^2 - 2\left(\frac{\langle\theta\rangle}{\langle s\rangle}\right)\eta_\omega^2, \tag{35}$$

where η_x^2, the total noise for *x*, can be expressed as

$$\eta_x^2 = \frac{Var(x)}{\langle x\rangle^2}, \tag{36}$$

which corresponds to CV^2, where *x* =*t*, θ, or ω. Results of such processing, with their CV^2 and mean signal, are included in Supplementary Fig. 6 below.

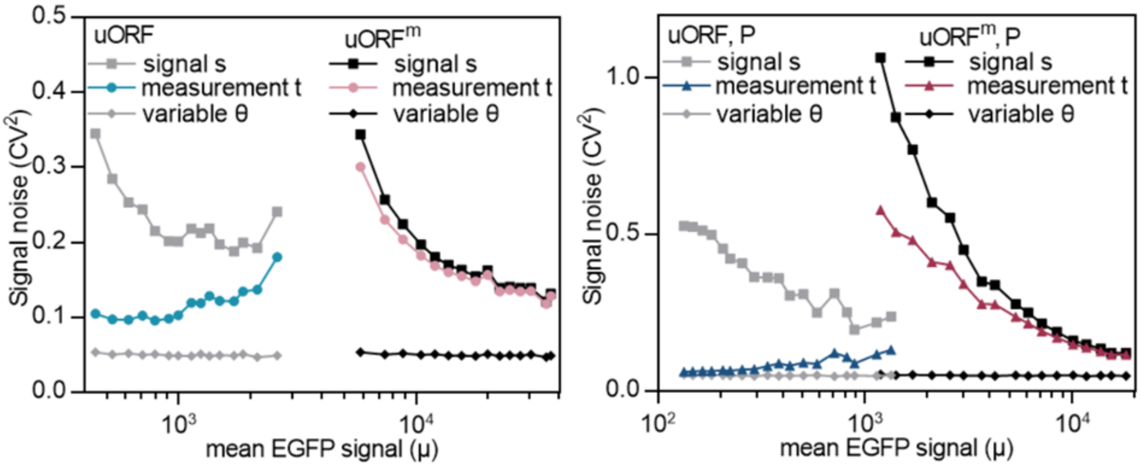

Supplementary Fig. 6 | Pairwise comparison of signal noise in fluorescence measurement t (protein native ●, PEST ▲), fluorescence signal s (■), and a random variable θ (◆).

In this study η_s^2 was obtained for the EGFP signals in the entire flow cytometry set of data, the subpopulations binned by mCherry in single uORF-EGFP or by the sum of mCherry and EGFP in cells with the dual uORF/uORF^m constructs. Square roots of η_s^2, or the CV (in %) are reported in Fig. 1 and Extended Data Fig. 1.

We also note that the linear calibration ($c = mx + b$) of fluorescence intensity for molecular numbers does not change the value of η_s^2 and other noise terms associated with signal s. Noise terms associated with t and θ are scaled by $(c\text{-}b)^2/c^2$, where c is the corresponding molecular counts and b is the intercept of the calibration.

Inferring intrinsic noise

We identified intrinsic noises from the dual uORF/uORF^m constructs for both GFP and mCherry by following the previous scheme[1]. In these experiments, the extrinsic noise is defined as[1,24]

$$\eta_{x,x'} = \frac{\mathrm{Cov}(x,x')}{\langle x\rangle\langle x'\rangle}, \tag{37}$$

where x and x' are the signals of GFP and mCherry in the dual uORF/uORF^m constructs. The intrinsic noise for signal s was evaluated as

$$I_{s|s'} = \frac{\langle t\rangle^2}{\langle s\rangle^2} I_{t|t'} - \frac{\langle\theta\rangle^2}{\langle s\rangle^2} I_{\theta|\theta'}, \tag{38}$$

where

$$I_{x|x'} = \eta_x^2 - \eta_{x,x'}, \tag{39}$$

is the intrinsic noise for x. Square roots of $I_{s|s'}$, are reported as the CV (in %) in Extended Data Fig. 2.

Analysis for single-cell trajectories

Determining the nuclear fluorescence signals

Single-cell trajectories are analyzed frame by frame, where each frame represents time points, which is taken every hour. The image size is 1,024×1,024 pixels, equivalent to 582×582 μm^2. The signals are stored in the format of unsigned 16-bit integer. The following are the four steps in our image analysis.

First, we use the Otsu method, with the package scikit-image[25] in python to find the edge of cells. The Otsu method[26] determines a threshold automatically, which separates total signals into two groups, with minimum variance in each group. Therefore, no manually adjusting parameter is needed. The edge of cells was found by a linear rescale to the lower group signals to 0-255, followed by a step to blur the image with the median value within the range of 3 pixels, and finally another Otsu method again to separate cells from the background. In this way, cells are in the segments with signals higher than the threshold, which is further constrained by the size being > 700 pixels, a fair lower bound of a living cell on the image. If the number of segments (cells) are not in the range of 50 to 120, we would lower the threshold before blurring by using the Otsu method on the lower signal group. Usually, by repeating the process 1–3 times, we can get a clear segmentation for most of the cells.

The second step is to separate the cells that are close to one another from the first step because their margins can be connected. We use the erosion method[27] several times to separate those cells when a single segment is > 5,400 pixels, a fair upper bound of the cell size. Erosion is repeated to separate the cells. The process will only stop when the largest remaining segment among the separated pieces is smaller than the size of 5,400 pixels being eroded the same number of times. The separated pieces are then dilated with the same number of erosion times to get a separated cell. Sometimes, going through the erosion too many time and dilate back to the same size, the segment's shape becomes diamond-shaped. However, such diamond shape is still within a cell, and the desirable nuclear signal is not affected.

The third step is tracking the same cell crossing different time frames. We overlapped the labeled cells between two consecutive frames. The largest overlapping area will indicate the same cell at different times. This method may not track cells that move much between two frames. However, there are very few such cells, and it seems that such moving cells are also vulnerable because they may lose the circular shape in a few frames.

Finally, we export the signals within the nuclei. We use the signal threshold value 20 to determine the region of nucleus within a cell. If the nucleus size is > 1800, then we speculate that the cell is dead and the nucleus size cannot be maintained. In addition, dead cell residue usually has high signals. Such residue may attach to a cell, causing the cell to always have high signals in all the frames; we also remove such tracking data.

Determining the peak phase in single-cell trajectories

To define the time for the peaks (the peak phase) in the trajectories, we used a Gaussian low-pass filter with a cut-off frequency of 0.05 (1/h), set as the standard deviation of the Gaussian filter function in the frequency domain. This is equivalent to preserving oscillations with periods of $\gtrsim$ 20 h in the original signal. The fast Fourier transform functions *fft* and *ifft* in the standard *numpy* module were used. With high-frequency components filtered out, the determination for the peaks as local maxima is straightforward. It was performed with the *find_peak* function from the *scipy* module, where a minimum distance between peaks was set to 15 h, and minimum height was set to 10 (with most of the peak values ranging from 20 to > 100). We subsequently determined the period length for each cell by taking the difference of two consecutive peak times. The peaks were further classified according to their peak phase time zones, with zone I being the beginning of observation to 20 h; zone II, 20-40 h, and zone III, after 40 h. For rhythmic trajectories, only cells with at least two peaks identified in two consecutive time zones were kept.

After this automated procedure, trajectories were further screened for the following possible artifacts: 1) the program might fail to track some cells afterwards, leading to zero signals in the latter frames; 2) the cell could die during the observation, leading to a very high fluorescence signal that does not oscillate; and 3) sometimes a piece of fast-moving, highly fluorescent debris or fragment of dead cells are captured, leading to a sudden up-and-down in the signal that remains quite isolated from other signals in time, and these are manually removed. Data shown in Fig. 4 and Extended Data Fig. 9, 10 are from data obtained in this process.

References cited in Supplementary Information

1 Schmiedel, J. M. *et al.* MicroRNA control of protein expression noise. *Science* **348**, 128-132, doi:10.1126/science.aaa1738 (2015).

2 Thattai, M. & van Oudenaarden, A. Intrinsic noise in gene regulatory networks. *P Natl Acad Sci USA* **98**, 8614-8619, doi:DOI 10.1073/pnas.151588598 (2001).

3 Mcquarri.Da. Stochastic Approach to Chemical Kinetics. *J Appl Probab* **4**,

413-478, doi:Doi 10.2307/3212214 (1967).
4 Gillespie, D. T. Exact Stochastic Simulation of Coupled Chemical-Reactions. *Abstr Pap Am Chem S* **173**, 128-128 (1977).
5 Gillespie, D. T. The chemical Langevin equation. *Journal of Chemical Physics* **113**, 297-306, doi:Doi 10.1063/1.481811 (2000).
6 Kampen, N. G. V. *Stochastic processes in physics and chemistry*. (North Holland, 2007).
7 Elf, J. & Ehrenberg, M. Fast evaluation of fluctuations in biochemical networks with the linear noise approximation. *Genome Research* **13**, 2475-2484, doi:10.1101/gr.1196503 (2003).
8 Paulsson, J. Summing up the noise in gene networks. *Nature* **427**, 415-418, doi:10.1038/nature02257 (2004).
9 Kurihara, Y. uORF Shuffling Fine-Tunes Gene Expression at a Deep Level of the Process. *Plants (Basel)* **9**, doi:10.3390/plants9050608 (2020).
10 Hinnebusch, A. G., Ivanov, I. P. & Sonenberg, N. Translational control by 5'-untranslated regions of eukaryotic mRNAs. *Science* **352**, 1413-1416, doi:10.1126/science.aad9868 (2016).
11 Ruiz-Echevarria, M. J. & Peltz, S. W. The RNA binding protein Pub1 modulates the stability of transcripts containing upstream open reading frames. *Cell* **101**, 741-751, doi:https://doi.org/10.1016/S0092-8674(00)80886-7 (2000).
12 Hastings, W. K. Monte-Carlo Sampling Methods Using Markov Chains and Their Applications. *Biometrika* **57**, 97-109, doi:DOI 10.1093/biomet/57.1.97 (1970).
13 Valderrama-Bahamóndez, G. I. & Fröhlich, H. MCMC Techniques for Parameter Estimation of ODE Based Models in Systems Biology. *Frontiers in Applied Mathematics and Statistics* **5**, doi:10.3389/fams.2019.00055 (2019).
14 Dempster, A. P., Laird, N. M. & Rubin, D. B. Maximum Likelihood from Incomplete Data via the EM Algorithm. *Journal of the Royal Statistical Society. Series B (Methodological)* **39**, 1-38 (1977).
15 Do, C. B. & Batzoglou, S. What is the expectation maximization algorithm? *Nature Biotechnology* **26**, 897-899, doi:10.1038/nbt1406 (2008).
16 Theodoridis, S. in 'Bayesian Learning: Inference and the EM Algorithm', Eds. *Machine Learning*, Sergios Theodoridis. *(Academic Press)*, 585-638 (2015).
17 Levine, R. A. & Casella, G. Implementations of the Monte Carlo EM algorithm. *J Comput Graph Stat* **10**, 422-439, doi:Doi 10.1198/106186001317115045 (2001).
18 Wu, F., Zilberstein, S. & Jennings, N. R. in *Proceedings of the Twenty-Third*

international joint conference on Artificial Intelligence 397–403 (AAAI Press, Beijing, China, 2013).

19 Corish, P. & Tyler-Smith, C. Attenuation of green fluorescent protein half-life in mammalian cells. *Protein Engineering, Design and Selection* **12**, 1035-1040, doi:10.1093/protein/12.12.1035 (1999).

20 Cai, L., Friedman, N. & Xie, X. S. Stochastic protein expression in individual cells at the single molecule level. *Nature* **440**, 358-362, doi:10.1038/nature04599 (2006).

21 Yan, C. S., Chepyala, S. R., Yen, C. M. & Hsu, C. P. Efficient and flexible implementation of Langevin simulation for gene burst production. *Sci Rep* **7**, 16851, doi:10.1038/s41598-017-16835-y (2017).

22 Metzl-Raz, E. *et al.* Principles of cellular resource allocation revealed by condition-dependent proteome profiling. *Elife* **6**, doi:10.7554/eLife.28034 (2017).

23 Heyer, E. E. & Moore, M. J. Redefining the translational status of 80S monosomes. *Cell* **164**, 757-769, doi:10.1016/j.cell.2016.01.003 (2016).

24 Elowitz, M. B., Levine, A. J., Siggia, E. D. & Swain, P. S. Stochastic gene expression in a single cell. *Science* **297**, 1183-1186, doi:DOI 10.1126/science.1070919 (2002).

25 van der Walt, S. *et al.* scikit-image: image processing in Python. *Peerj* **2**, e453, doi:10.7717/peerj.453 (2014).

26 Otsu, N. A threshold selection method from gray-level histograms. *IEEE Transactions on Systems, Man, and Cybernetics* **9**, 62-66 (1979).

27 Serra, J. *Image analysis and mathematical morphology*. (Academic Press, Inc., 1983).

Supplementary Table 1 Estimated parameters of the reaction rates in the translation models

Meaning			Replicate 1		Replicate 2		Replicate 3	
**Dual fluorescence assay**		**Eq***	**Regular**	**uORF-mediated**	**Regular**	**uORF-mediated**	**Regular**	**uORF-mediated**
k_m#	Transcription/RNA production	1, 10	0.06295		0.04252		0.05738	
γ_{mF}/ γ_{muF}	Free-form RNA degradation	1, 10	0.2521		0.2773		0.2773	
k_P	Translation/protein production	2	1290000	-	3555000	-	1938000	-
γ_{mu}	uORF-occupied RNA degradation	11	-	0.393	-	0.265	-	0.1944
k_{on}	Ribosome associated with uORF	10	-	3096000	-	19230000	-	140100000
k_{off}	Ribosome dissociated from uORF	10	-	0.5835	-	0.09216	-	0.2457
k_{Pu}	Translational reinitiation/ protein production	10	-	194000	-	243500	-	109500
γ_P	Protein degradation	2, 12	0.02666		0.02567		0.02567	
γ_{P-PEST}	Protein degradation with PEST-mediated destabilization	2, 12	0.08381		0.0829		0.06918	

*: The first appearance of the reaction rate in reactions in Supplementary Information (page 1-3).
#: k_m is in the unit of molecule number/time per cell, while all the other rates are first-order reaction rates with units of 1/time. We adopt the molecule number calibration and time, arbitrarily, since we only fit for the steady state.

Supplementary Table 2 Primers used in this study.

DNA fragment	Oligo name	Sequence (5’ to 3’)
**Constructs for protoplast transient assays**		
LexA operon-35S promoter	Op. LexA-*Hind*III-Fw	TATAAGCTTGGGCTGCAGGTCGAGG
	p35S-*Xba*I, *Bam*HI-Rev	CTTGGATCCGATATCTAGACTAGCTTCAGCGTGTCC
EGFP-ZmSP	*EGFP-Bam*HI-Fw	GAGGATCCAACAATGGTGAGCAAGGG
	EGFP-ZmSP-Rev	TATGCTTCCAGTTCTCTTCTCTTGTACAGCTCGTCCATGC
	EGFP-ZmSP-Fw	GCATGGACGAGCTGTACAAGAGAAGAGAACTGGAAGCATA
	ZmSP-*Sac*I-Rev	TTCGAGCTCTTAGGAAGCTGTAAGAATGGCGTTG
EGFP	*EGFP-Sac*I-Rev	TGGAGCTCTTACTTGTACAGCTCGTC
T_{Nos}-O_{LexA}-*MCS*-T_{3A}	Ter. Nos-*Sac*I-Fw	TAAGAGCTCATGATCCCCGATCGTTCAAAC
	Ter. 3A-*Eco*RI-Rev	AGTGAATTCCTCGACTCGGTACCCCCTCGACAC
mCherry-ZmSP	*mCherry-Xho*I-Fw	AACTCGAGATGGTGAGCAAGGGCGAGGA
	*mCherry-Bam*HI-Fw	GCAGGATCCAACAATGGTGAGCAAGGGCGAGGA
	mCherry-ZmSP-Rev	TATGCTTCCAGTTCTCTTCTCTTGTACAGCTCGTCCATGC
	mCherry-ZmSP-Fw	GCATGGACGAGCTGTACAAGAGAAGAGAACTGGAAGCATA
	ZmSP-*Spe*I-Rev	CCACTAGTTTAGGAAGCTGTAAGAATGGCGT
mCherry	*mCherry-Spe*I-Rev	CCACTAGTTTACTTGTACAGCTCGTCCATGC
**Site-directed mutagenesis of *AtFKBP20-1* 5' leader**		
AtFKBP20-1 5' leader (AUG-uORF)	*FKBP20*-5’L-*Xba*I-Fw	AGATCTAGAATAACACGTGACGTCAA
	FKBP20-5’L-*Xho*I-Fw	AGCCTCGAGAATAACACGTGACGTCAAAA
	FKBP20-5’L-*Bam*HI-Rev	ATTGGATCCAGCTTCAAAGGTTCAAAG
AtFKBP20-1 5' leader (mutated CUG-uORF)	*FKBP20*-CTG-Fw	TTTGGACAGTCTcTGGCCACCGATC
	FKBP20-CTG-Rev	GATCGGTGGCCAgAGACTGTCCAAA
**Site-directed mutagenesis *TOC1* 5' leader**		
TOC1 5' leader (AUG-uORF)	*TOC1*-5'L-*Xba*I-Fw	TATCTAGACGTAAGAAAAAGACTTAATCTC
	TOC1-5'L-*Bam*HI-Rev	TATGGATCCGATCAGATTAACAACTAAACC
TOC1 5' leader (mutated stop-uORF)	*TOC1*-1-TAG-Fw	GATTGACAAAACAAAACAtagAGTCTTCTTTCTCTTCTC
	TOC1-1-TAG-Rev	GAGAAGAGAAAGAAGACTctaTGTTTTGTTTTGTCAATC
	TOC1-2-TGA-*Eco*RI-F	GAGCTTTTTTGTTGATTCtgaATTCGATATAGAGAAAAAAGA
	TOC1-2-TGA-*Eco*RI-R	TCTTTTTTCTCTATATCGAATtcaGAATCAACAAAAAAGCTC
	TOC1-3-TAG-Fw	TATCTTTTTGTTTGTTCTTtagGATAAAGGATTAGGTTG
	TOC1-3-TAG-Rev	CAACCTAATCCTTTATCctaAAGAACAAACAAAAAGATA
	TOC1-4-TAG-Fw	CCTAGTTCTGATTTGGCCtagGAAGTATCATAGCACTTAC
	TOC1-4-TAG-Rev	GTAAGTGCTATGATACTTCctaGGCCAAATCAGAACTAGG
**qRT-PCR**		
TOC1	*TOC1*-qPCR-Fw	CTTACTACCACCACCCAATGAACAC
	TOC1-qPCR-Rev	TGAACCATAGACATCTGACCATTCTG
UBQ10	*UBQ10*-qPCR-Fw	AGAAGTTCAATGTTTCGTTTCATGTAA
	UBQ10-qPCR-Rev	GAACGGAAACATAGTAGAACACTTATTCA

EGFP	*eGFP*-qPCR-Fw	GCACAAGCTGGAGTACAACTA
	eGFP-qPCR-Rev	TGTTGTGGCGGATCTTGAA
mCherry	*mCherry*-qPCR-Fw	CGAGATCAAGCAGAGGCTGAA
	mCherry-qPCR-Rev	TCTTGACCTCAGCGTCGTAGTG
