# Supplementary material for: Noise reduction by upstream open reading frames
Source: Nat Plants. 2022 May 2;8(5):474–80. doi: 10.1038/s41477-022-01136-8 (PMC9122824; doi:10.1038/s41477-022-01136-8)
Supplement: Supplementary file 2 — Reporting Summary [file 41477_2022_1136_MOESM2_ESM.pdf]

nature research

Corresponding author(s): Shu-Hsing Wu and Chao-Ping Hsu

Last updated by author(s): 2022/03/05

Reporting Summary

Nature Research wishes to improve the reproducibility of the work that we publish. This form provides structure for consistency and transparency in reporting. For further information on Nature Research policies, see our Editorial Policies and the Editorial Policy Checklist.

Statistics

For all statistical analyses, confirm that the following items are present in the figure legend, table legend, main text, or Methods section.

n/a	Confirmed	
☐	☒	The exact sample size (*n*) for each experimental group/condition, given as a discrete number and unit of measurement
☐	☒	A statement on whether measurements were taken from distinct samples or whether the same sample was measured repeatedly
☐	☒	The statistical test(s) used AND whether they are one- or two-sided<br>*Only common tests should be described solely by name; describe more complex techniques in the Methods section.*
☐	☒	A description of all covariates tested
☐	☒	A description of any assumptions or corrections, such as tests of normality and adjustment for multiple comparisons
☐	☒	A full description of the statistical parameters including central tendency (e.g. means) or other basic estimates (e.g. regression coefficient) AND variation (e.g. standard deviation) or associated estimates of uncertainty (e.g. confidence intervals)
☐	☒	For null hypothesis testing, the test statistic (e.g. *F*, *t*, *r*) with confidence intervals, effect sizes, degrees of freedom and *P* value noted<br>*Give P values as exact values whenever suitable.*
☐	☒	For Bayesian analysis, information on the choice of priors and Markov chain Monte Carlo settings
☒	☐	For hierarchical and complex designs, identification of the appropriate level for tests and full reporting of outcomes
☒	☐	Estimates of effect sizes (e.g. Cohen's *d*, Pearson's *r*), indicating how they were calculated

Our web collection on statistics for biologists contains articles on many of the points above.

Software and code

Policy information about availability of computer code

Data collection	Attune NxT Software v2.7 and v3.1, LightField v6.10, QuantStudio™ 12K Flex Software v1.2.2, VisionWorks v9.1.20063.7760, Leica Application Suite X_4.1.0.23081.
Data analysis	FlowJo v10, ImagePro v6.2, BRASS v3.0, Fiji_win64, QuantStudio™ 12K Flex Software v1.4, VisionWorks v9.1.20063.7760, R x64 4.0.3, additional customized code deposited in GitHub web-link, https://github.com/cherrihsu/uORF_dataprocess.

For manuscripts utilizing custom algorithms or software that are central to the research but not yet described in published literature, software must be made available to editors and reviewers. We strongly encourage code deposition in a community repository (e.g. GitHub). See the Nature Research guidelines for submitting code & software for further information.

Data

Policy information about availability of data

All manuscripts must include a data availability statement. This statement should provide the following information, where applicable:

- Accession codes, unique identifiers, or web links for publicly available datasets
- A list of figures that have associated raw data
- A description of any restrictions on data availability

Data availability statement is included in the manuscript. Data associated with figures/ED figures have been made available as Source Data.

Field-specific reporting

Please select the one below that is the best fit for your research. If you are not sure, read the appropriate sections before making your selection.

☒ Life sciences ☐ Behavioural & social sciences ☐ Ecological, evolutionary & environmental sciences

For a reference copy of the document with all sections, see nature.com/documents/nr-reporting-summary-flat.pdf

Life sciences study design

All studies must disclose on these points even when the disclosure is negative.

Sample size	No sample-size calculation performed. At least three independent experiments performed as a common practice.
Data exclusions	In dual fluorescence quantification experiments, in each binning, fluorescence levels above 99% and below 1% were considered outliers and removed. No data were excluded in uORF-TOC1 and uORFm-TOC1 experiments.
Replication	At least three independent biological replicates were performed in all the experiments described.
Randomization	Quantification of RNA levels, protein levels, and period lengths were randomized for experiments involving TOC1 transgenic plants in results shown in Fig. 3 and Extended Data Figure 4, 5, 6, 7.
Blinding	Quantification of RNA levels, protein levels, and period lengths were blind-folded for experiments involving TOC1 transgenic plants in results shown in Fig. 3 and Extended Data Figure 4, 5, 6, 7. Blind test is irrelevant for flow data (Fig. 1) and time-course imaging (Fig. 4) with automatic data acquisition.

Reporting for specific materials, systems and methods

We require information from authors about some types of materials, experimental systems and methods used in many studies. Here, indicate whether each material, system or method listed is relevant to your study. If you are not sure if a list item applies to your research, read the appropriate section before selecting a response.

Materials & experimental systems

n/a	Involved in the study	
☐	☒	Antibodies
☒	☐	Eukaryotic cell lines
☒	☐	Palaeontology and archaeology
☒	☐	Animals and other organisms
☒	☐	Human research participants
☒	☐	Clinical data
☒	☐	Dual use research of concern

Methods

n/a	Involved in the study	
☒	☐	ChIP-seq
☐	☒	Flow cytometry
☒	☐	MRI-based neuroimaging

Antibodies

Antibodies used	anti-eGFP (GenLab, CAT#50005-05), LexA (Abcam, ab14553).
Validation	No cross recognition of endogenous proteins near the molecular weight of the protein of interests (TOC1-YFP and XVE) via immunoblot analyses in our parental line samples.

Flow Cytometry

Plots

Confirm that:

☒ The axis labels state the marker and fluorochrome used (e.g. CD4-FITC).

☒ The axis scales are clearly visible. Include numbers along axes only for bottom left plot of group (a 'group' is an analysis of identical markers).

☒ All plots are contour plots with outliers or pseudocolor plots.

☒ A numerical value for number of cells or percentage (with statistics) is provided.

Methodology

Sample preparation	Mesophyll protoplasts were freshly prepared from 4-week-old transgenic Arabidopsis plants grown under 12-h light/12-h dark at 22℃) and transfected with constructs listed prior to flow cytometry analyses.
Instrument	Attune NxT Flow Cytometer (ThermoFisher)
Software	Attune NxT Software v2.7 and v3.1, FlowJo v10
Cell population abundance	The purity of the sample is confirmed by gating strategy and inspection of autofluorescence. The post-sort cell abundance is 3,600-27,000 cells.
Gating strategy	FSC-H/SSC-H gates the protoplast population as starting cell population. EGFP and mCherry fluorescence thresholds were determined by eliminating signals for 99.9% cells in protoplasts expressing mCherry-only and EGFP-only, respectively.

☒ Tick this box to confirm that a figure exemplifying the gating strategy is provided in the Supplementary Information.
