# Supplementary figures and images for: Noise reduction by upstream open reading frames
Source: Nat Plants. 2022 May 2;8(5):474–80. doi: 10.1038/s41477-022-01136-8 (PMC9122824; doi:10.1038/s41477-022-01136-8)

Full-sized images for results shown in Extended Data Fig. 5a.

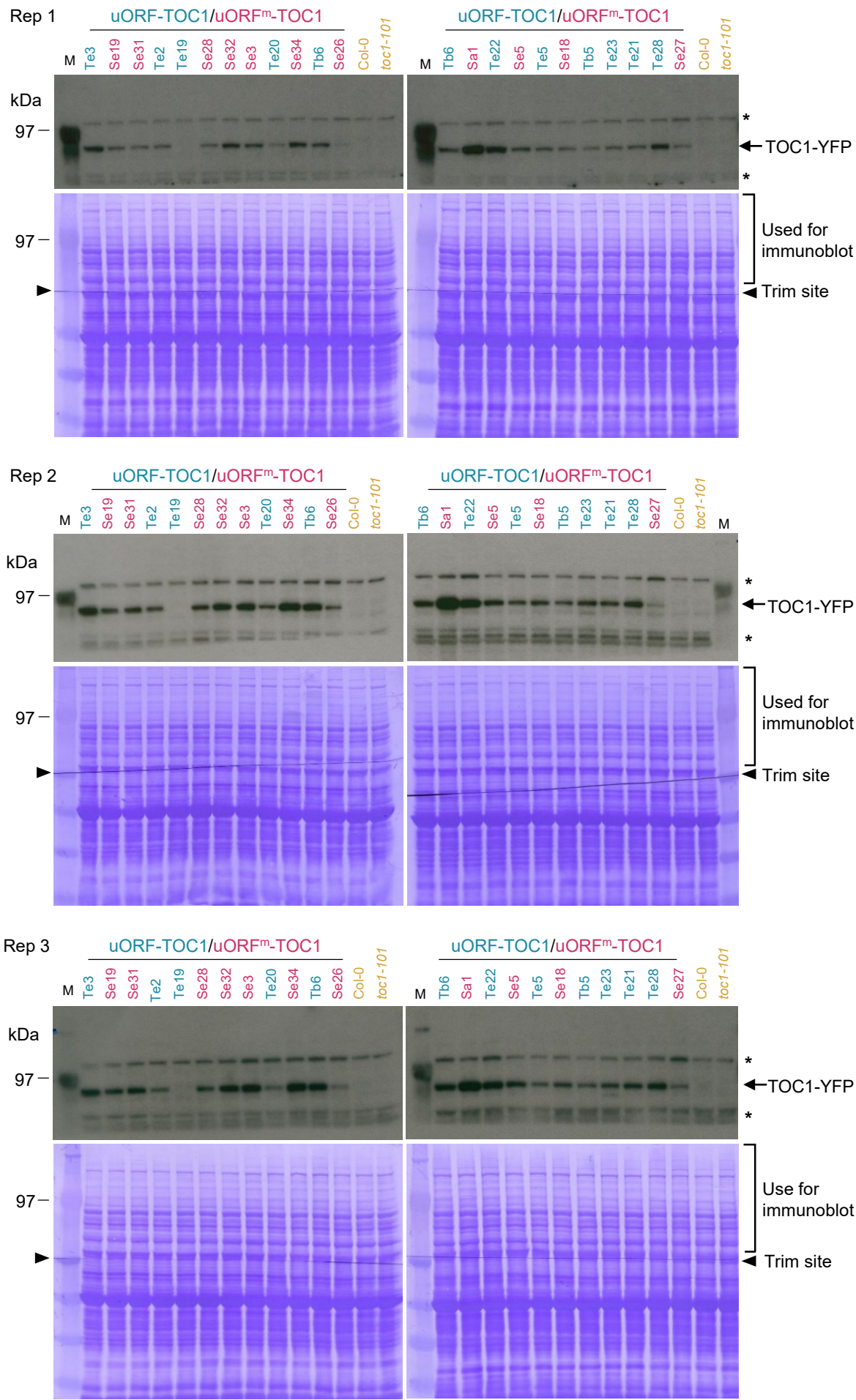

Supplement: Source Data Extended Data Fig. 5 — Unprocessed blots and gels for Extended Data Fig.5a. [file 41477_2022_1136_MOESM9_ESM.pdf]

Full-sized images for results shown in Extended Data Fig. 8b.

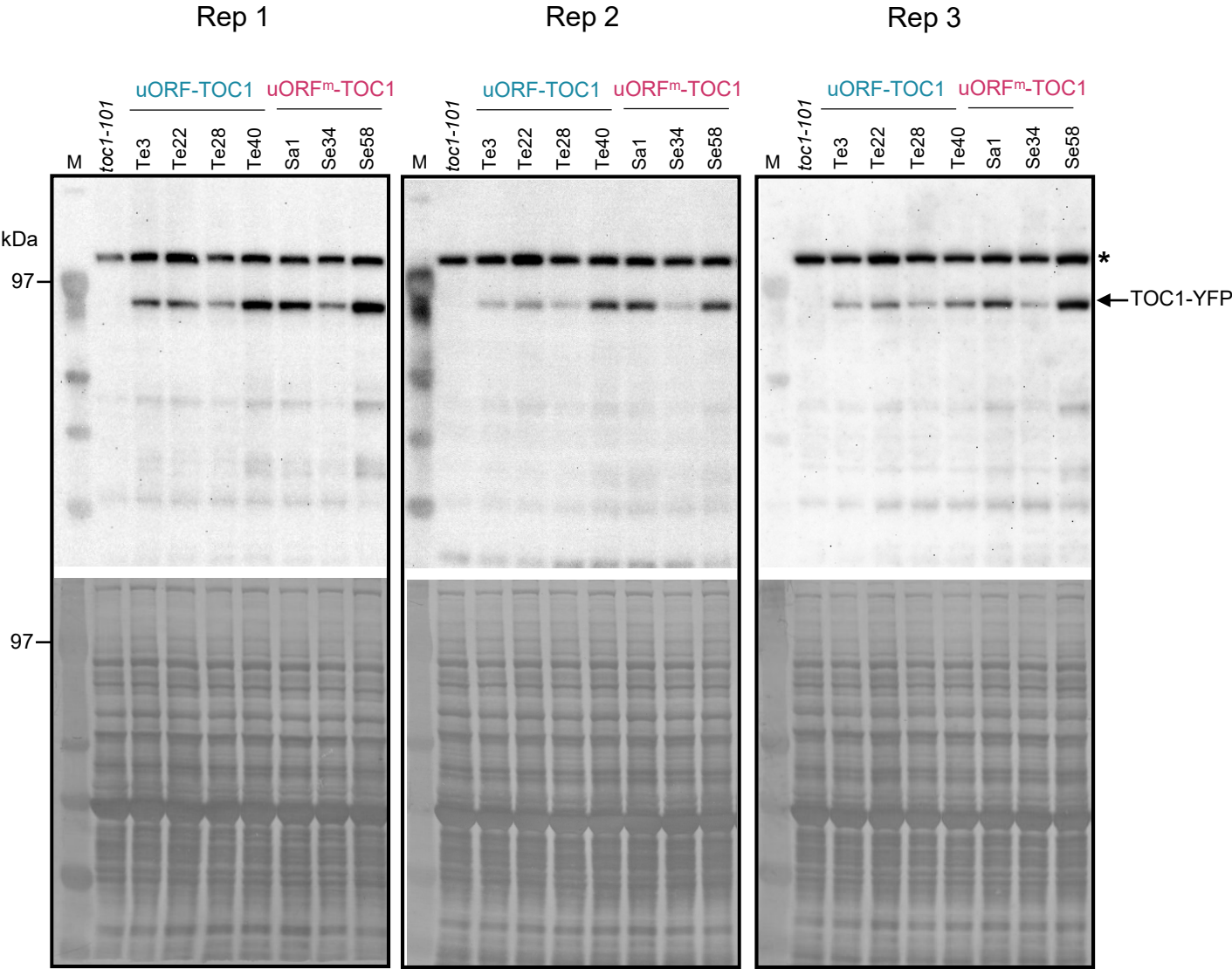

Supplement: Source Data Extended Data Fig. 8 — Unprocessed blots and gels for Extended Data Fig. 8b. [file 41477_2022_1136_MOESM11_ESM.pdf]
